# Native ESI Mass Spectrometry Can Help to Avoid Wrong Interpretations from Isothermal Titration Calorimetry in Difficult Situations

**DOI:** 10.1007/s13361-016-1534-6

**Published:** 2016-12-12

**Authors:** Philippe Wolff, Cyrielle Da Veiga, Eric Ennifar, Guillaume Bec, Gilles Guichard, Dominique Burnouf, Philippe Dumas

**Affiliations:** 1 Biophysics and Structural Biology Team, Unité Architecture et réactivité de l’ARN (UPR9002), Institut de Biologie Moléculaire et Cellulaire du CNRS, Université de Strasbourg, 15, rue René Descartes, F67084 Strasbourg cedex, France; 2 Plateforme protéomique Strasbourg-Esplanade, Institut de Biologie Moléculaire et Cellulaire du CNRS, FRC 1589, Université de Strasbourg, 15, rue René Descartes, F67084 Strasbourg cedex, France; 3 CBMN, UMR 5248, Institut Européen de Chimie et Biologie, Université de Bordeaux, 2 rue Robert Escarpit, 33607 Pessac, France; 4 CNRS, CBMN, UMR 5248, 33600 Pessac, France; 5 Institut de Biologie et Génétique Moléculaire, Université de Strasbourg, 1, rue Laurent Fries, 67400 Illkirch, France

**Keywords:** DNA-polymerase processivity rings, K_d_ determination, ESI-MS, ITC

## Abstract

We studied by native ESI-MS the binding of various DNA-polymerase-derived peptides onto DNA-polymerase processivity rings from *Escherichia coli*, *Pseudomonas aeruginosa*, and *Mycobacterium tuberculosis*. These homodimeric rings present two equivalent specific binding sites, which leads to successive formation during a titration experiment of singly- and doubly occupied rings. By using the ESI-MS free-ring spectrum as a ruler, we derived by robust linear regression the fractions of the different ring species at each step of a titration experiment. These results led to accurate K_d_ values (from 0.03 to 0.5 μM) along with the probability of peptide loss due to gas phase dissociation (GPD). We show that this good quality is due to the increased information content of a titration experiment with a homodimer. Isothermal titration calorimetry (ITC) led with the same binding model to K_d_(ITC) values systematically higher than their ESI-MS counterparts and, often, to poor fit of the ITC curves. A processing with two competing modes of binding on the same site requiring determination of two (K_d_, ΔH) pairs greatly improved the fits and yielded a second K_d_(ITC) close to K_d_(ESI-MS). The striking features are: (1) ITC detected a minor binding mode (~20%) of ‘low-affinity’ that did not appear with ESI-MS; (2) the simplest processing of ITC data with only one (K_d_, ΔH) pair led wrongly to the Kd of the low-affinity binding mode but to the ΔH of the high-affinity binding mode. Analogous misleading results might well exist in published data based on ITC experiments.

**Electronic supplementary material:**

The online version of this article (doi:10.1007/s13361-016-1534-6) contains supplementary material, which is available to authorized users.

**Graphical Abstract:**
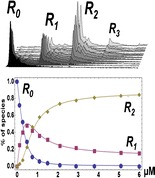
ᅟ

## Introduction

Mass spectrometry using electrospray ionization (ESI-MS) in native conditions has been pioneered in 1991 by two almost simultaneous studies, one on the observation of enzyme–substrate and enzyme–product complexes [1] and another one on the observation of the heme–globin complex in native hemoglobin [2]. Since then, ESI-MS in native conditions has become common usage for studying noncovalent biological complexes [3]. However, a major problem attached to this approach is the possible partial dissociation of the complex following the transfer into the gas phase. This is known as gas-phase dissociation (GPD), first studied quantitatively in [4]. This is a major practical problem because in the absence of additional information, it is difficult to disentangle the amount of GPD from the determination of the dissociation constant *K*_*d*_ characterizing the interaction. This difficulty stems from the fact that by ignoring the existence of GPD, an apparent *K*_*d*_^*app*^ may often account reasonably for the experimental data from a titration experiment (see section ‘*To which extent can a single K*_*d*_ *explain data affected by GPD?*’ in Supplementary Data for a quantitative discussion of this problem). In any case, an increase of GPD leads to an apparent affinity lower than the real one (hence to *K*_*d*_^*app*^ higher than *K*_*d*_). This becomes crucial due to the so-called ‘aggregation problem,’ which is an opposite effect resulting from the strengthening of nonspecific electrostatic interactions in the gas phase. This was particularly clear with positively charged antibiotics interacting with RNA [5]. It is then quite possible that these nonspecific and irrelevant interactions can be mistakenly considered as genuine specific interactions, particularly if the latter were thought to be lowered by GPD. In the field of drug development, this may become a serious problem, particularly when searching for a lead compound that is expected to bind poorly. Several lines of investigation have been undertaken, either to minimize the problem or to take it into account at the data-processing stage. Minimization of GPD has been obtained by using additives such as imidazole or sulfur hexafluoride in the gas phase [6, 7]. The favorable effect preserving the integrity of the complex of interest is thought to be due to evaporative cooling of the additive evacuating extra energy from the collisions with the neutral-gas molecules. Another possibility is the use of infrared radiation to heat the molecular complexes in the gas phase and dissociate nonspecific interactions [8]. However, a sufficient difference in stability of the specific and nonspecific interactions is necessary to avoid dissociating significantly the specific interactions too. Another method, not for minimizing GPD but to quantify it, is to introduce both the protein of interest and an unrelated protein as a reporter for aggregation [9–11]. The latter method was thought to be misleading for aggregation resulting from strong electrostatic interactions and was thus modified by using a reference cognate ligand competing with a putative ligand for the same site [12]. Finally, a processing method taking into account directly the amount of GPD (in conjunction with a micro gel filtration step to remove excess of ligand) was proposed [13].

Here we show that with a dimeric protein with two equivalent and independent binding sites, the previously mentioned problems resulting from the correlation between the *K*_*d*_ and the amount of GPD are considerably minimized for two reasons. First, instead of only one independent titration curve to be explained (fraction of complex versus concentration of the ligand), there are now two independent experimental curves to be explained (fractions of singly- and doubly occupied dimers versus concentration of the ligand), which represents an important increase of experimental information. Furthermore, there are no additional free parameters to be adjusted to account for this 2-fold amount of information. As a consequence, contrary to the previous situation, it is now mathematically impossible to obtain a single *K*_*d*_^*app*^ accounting for the two independent experimental curves if one ignores the existence of GPD. In fact, it may be shown that there is a mathematical solution for *K*_*d*_^*app*^ explaining the evolution of the fraction of singly occupied dimers, but it often leads to absurd negative values, and there is no solution at all explaining the evolution of the fraction of doubly occupied dimers. As a result, the important correlation between *K*_*d*_ and GPD with a monomeric macromolecule disappears completely with a dimer having two identical and independent binding sites. We show in the following that not only the *K*_*d*_ for the specific protein/ligand interaction and the amount of GPD but also (in one case) a *K*_*d*_ **-**like value for nonspecific aggregation could be determined accurately. Importantly, this was achieved without recourse to additional experimental steps such as the use of (1) gas additives, (2) a reference protein (or ligand), and/or (3) a chromatographic step prior to injection in the instrument.

**Figure 1:**
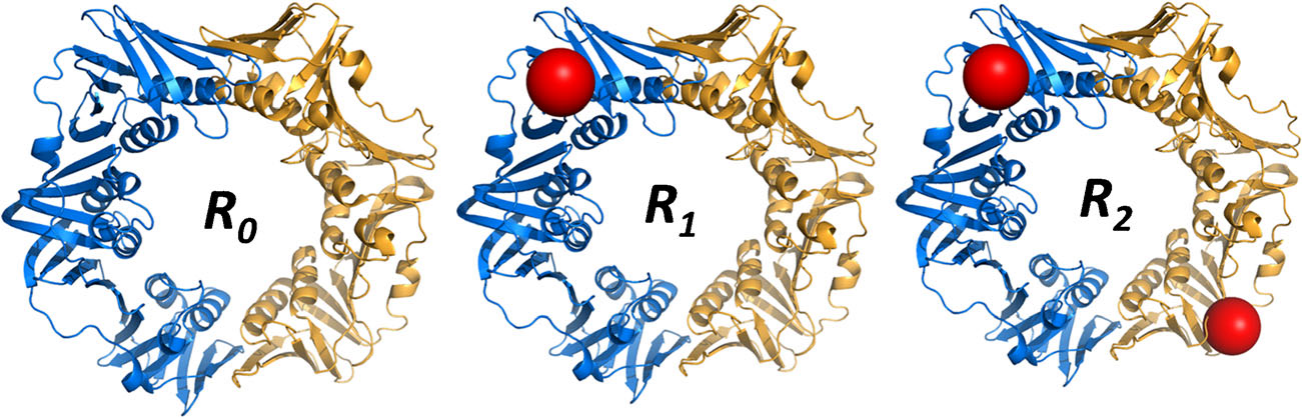
Illustration of the ring species *R*_*0*_, *R*_*1*_ and *R*_*2*_ *.* Structure of the *E.coli* processivity factor [13]. Each specifically-bound peptide is represented as a sphere at the position known from X-ray crystallography [16–18]. Nonspecific binding site(s) for a third peptide discussed in the following is (are) not known

In the frame of an ongoing study on the search for new antibiotics, we focus in this work on the interaction of three synthetic peptide ligands (Supplementary Figure S2) with three DNA-polymerase processivity factors (β-rings) from bacterial origin (*Escherichia coli*, *Mycobacterium tuberculosis*, and *Pseudomonas aeruginosa*) [14, 15]. These β-rings are homodimers (Figure 1) that bind specifically the ligands [16, 17]. The synthetic peptides were derived from the natural peptides that are present in each DNA-polymerase and that mediate the DNA-polymerase/β-ring interaction [18].

## Experimental

### Protein Production and Purification

The three *dnaN* bacterial genes were cloned into pET15b plasmid (Invitrogen, Carlsbad, CA, USA) using standard protocols. The resulting N-tagged protein was expressed in BL21 *E. coli* cells after IPTG induction (0.1 mM) at 28 °C. The β protein fractions were first enriched on a Ni-NTA column, eluted with a histidine step (300 mM), and further purified on a MonoQ column in buffer containing 20 mM Tris HCl pH 7.5, 0.5 mM EDTA, and 10% glycerol, using a gradient from 0 to 0.5 M NaCl. The purified proteins were dialyzed and concentrated (around 300 μM) against buffer containing 20 mM Tris HCl pH 7.5, 0.5 mM EDTA, and 10% glycerol.

### Peptide Synthesis

Procedures for the synthesis of the different peptides used in this study (**P6**, **P11**, and **P14**) have been described elsewhere [17]. The chemical-structure formulae of the peptides are shown in Supplementary Figure S2.

### Mass Spectrometry Measurements

Mass spectrometry studies were performed on a Micromass Q-Tof Micro (Waters Milford, MA, USA). ESI conditions were optimized to preserve the noncovalent complexes during the ionization-desorption process. To reach sufficient desolvation while minimizing dissociation of the noncovalent dimers, accelerating cone voltage was tuned to 100 V, extraction cone to 30 V, and desolvation temperature to 150 °C [19]. The pressure in the interface region was increased by 2.4 to 4.5 mbar to increase the signal-to-noise ratio [20]. Data were acquired in positive ion mode and accumulated during 3 min for each sample.

### ESI-MS Titration Experiments

Peptide stock solutions were prepared by dissolving peptides to 3.8 mM in 100% DMSO. For all titration experiments, the different proteins were at a constant monomer concentration of 0.6 μM in 50 mM ammonium acetate, pH 7. Peptides were added at 22.5 °C just before injection to avoid aggregation. The final DMSO concentration was maintained to 1% (v/v) to keep the same protein charge state for each titration point. Table 1 gives the concentrations used in the titration experiments.

**Table 1:** Protein and Peptide Characteristics

*E. coli*	43286	**P6**	676.8	0.12, 0.28, 0.49, 0.75, 1.1, 1.5, 2.1, 2.9, 3.8, 5.1, 6.0
		**P11**	730.4	0.08, 0.18, 0.31, 0.47, 0.67, 0.93, 1.3, 1.7, 2.2, 2.8, 3.6, 4.7
		**P14**	785.7	0.03, 0.06, 0.1, 0.15, 0.2, 0.28, 0.36, 0.46, 0.59, 0.74, 0.92, 1.4, 2.0
*M. tuberculosis*	44276	**P14**	785.7	0.12, 0.28, 0.49, 0.75, 1.1, 1.5, 2.1, 2.9, 3.8, 5.1, 6.0
*P. aeruginosa*	42727	**P11**	730.4	0.08, 0.18, 0.31, 0.47, 0.67, 0.93, 1.3, 1.7, 2.2, 2.8, 3.6, 4.7
		**P14**	785.7	0.03, 0.06, 0.1, 0.15, 0.2, 0.28, 0.36, 0.46, 0.59, 0.74, 0.92, 1.4, 2.0

The molecular weights (g mol^−1^) of the protein monomers (col. 2) and of the peptides **P6**, **P11**, and **P14** (col. 4) are indicated. Last column: peptide concentrations (μM) used for each titration

### ESI-MS Data Processing

**Figure 2:**
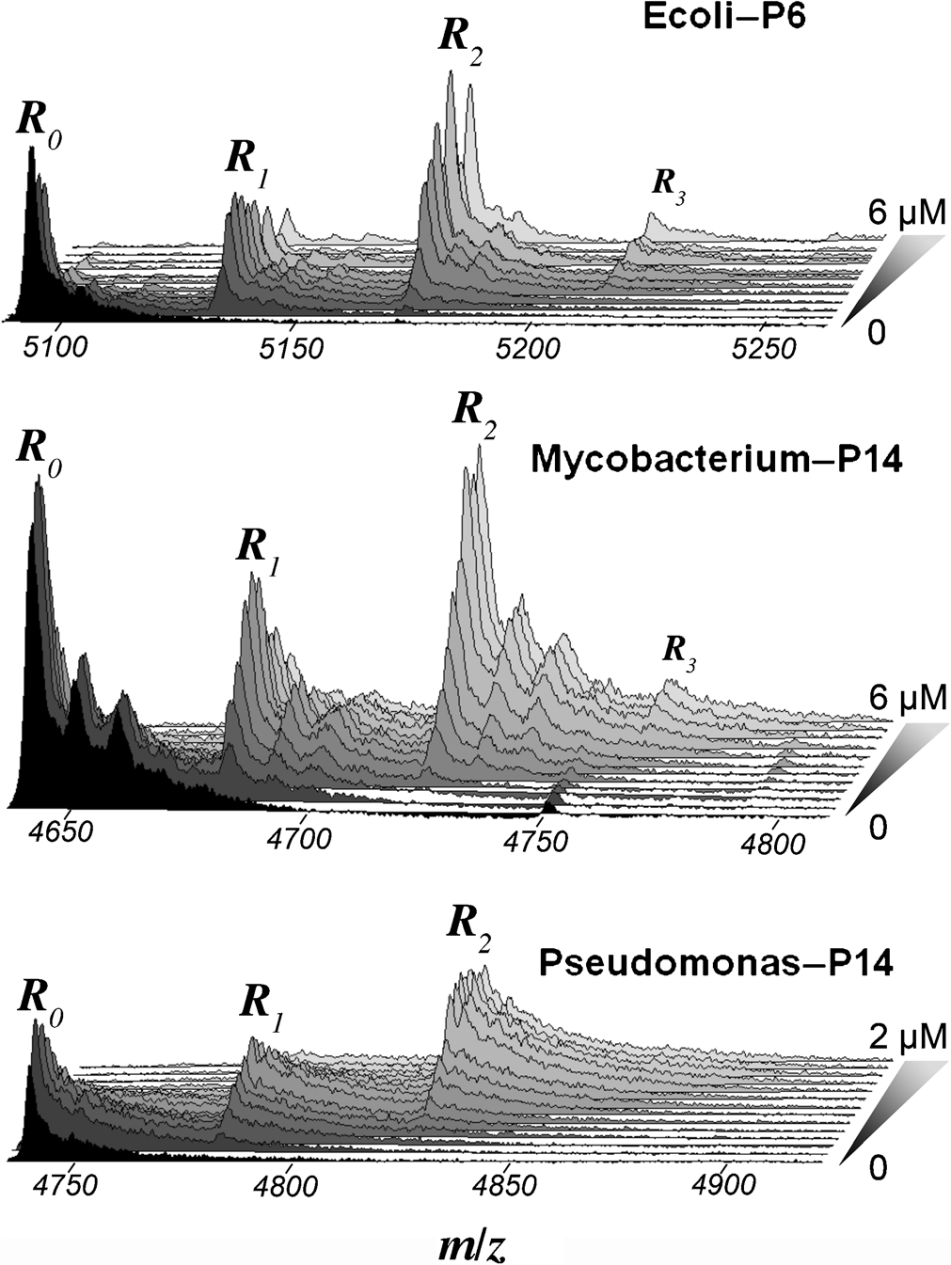
Raw experimental ESI-MS spectra for selected protein-peptide titrations. The spectra for all peptide concentrations used in each titration experiment are stacked to highlight the evolution of the populations of the species *R*_0_, *R*_1_, *R*_2_ with increasing peptide concentrations (indicated on the right). The charge states for the selected spectra are +17, +19, and +18 for *E. coli*, *Mycobacterium* and *Pseudomonas*, respectively. For β_*E.coli*_/**P6** and to a lesser extent for β_*Mycobacterium*_ */* **P14**, nonspecific weak binding of a third peptide is clearly visible (*R*_3_). The rather noisy satellite peaks appearing on the tails of *R*_0_, *R*_1_, *R*_2_ for *E. coli* **–P6** correspond to one and two DMSO adducts and for β_*Mycobacterium*_ */* **P14**, these peaks stand out more prominently and most likely correspond to two and four DMSO adducts. The remaining spectra for β_*E.coli*_/**P11**, β_*E.coli*_/**P14**, β_*Pseudomonas*_/**P11** are in Supplementary Figure S3

**Figure 3:**
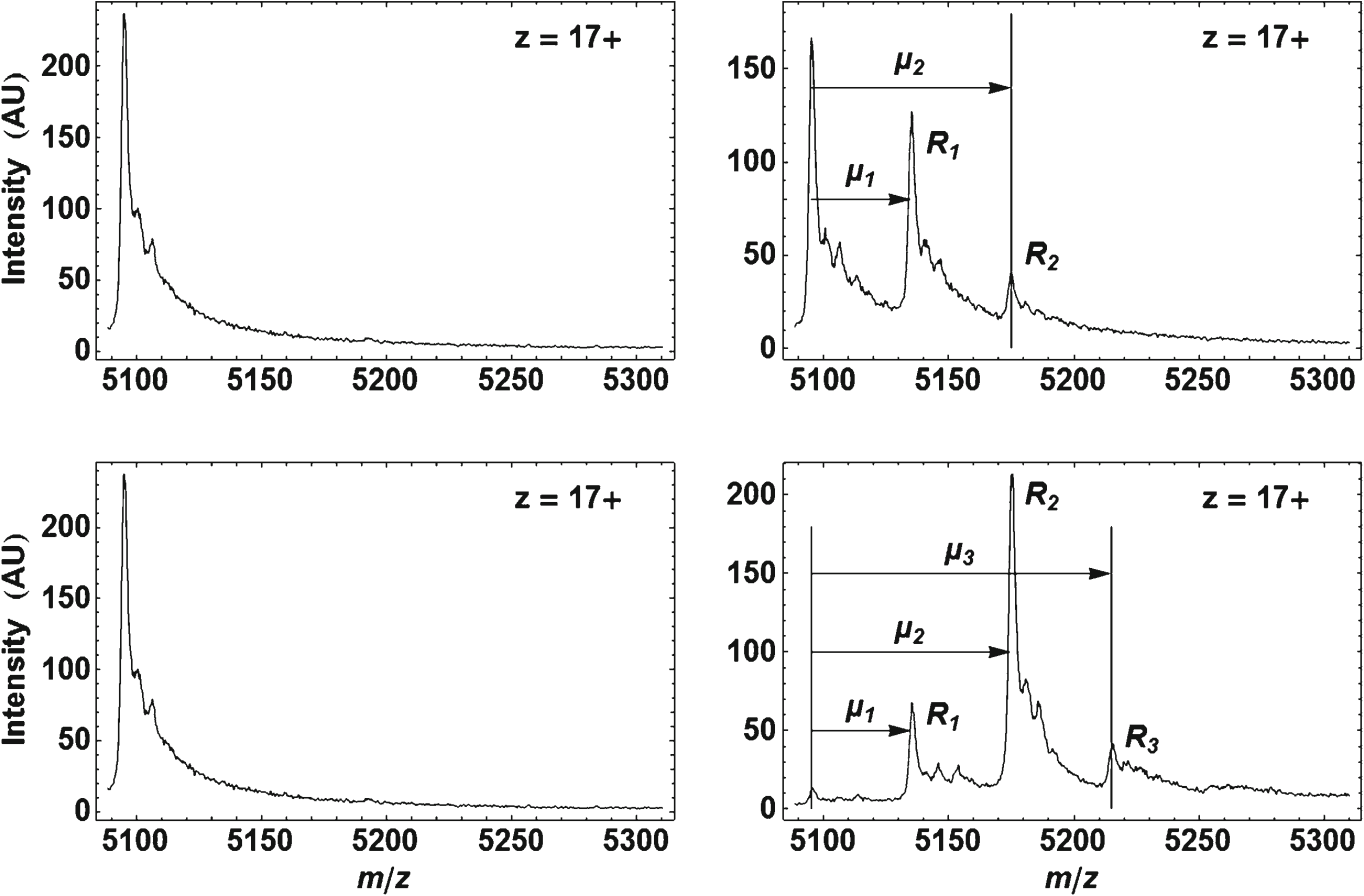
Illustration of the parameters in Equation 1 with β_*E.coli*_/P6 spectra. Left column: same reference spectrum *s*_0_(*m*/*z*) from the free-ring species *R*_0_ at **z** = +17. Right column: corresponding spectra *s*_0,1,2_(*m*/*z*) for *R*_0_ + *R*_1_ + *R*_2_ formed at [P6] = 0.28 μM (top) and *s*_0,1,2,3_(*m*/*z*) for *R*_0_ + *R*_1_ + *R*_2_ + *R*_3_ formed at [**P6**] = 2.9 μM (bottom). The arrows highlight the *m/z* shifts *µ*_1_, *µ*_2_ and *µ*_3_ mentioned in Equation 1. See Figure 4 for all spectra obtained during the whole titration

Raw spectra were obtained as Excel files and processed entirely with our own programs developed with *Mathematica* from Wolfram Research. The data were first smoothed with a moving Gaussian filter on 30 successive points. Since the protein monomers are tightly associated as dimeric rings, there are two specific peptide binding sites on each ring (Figure 1). As a consequence, we always observed at least three types of complexes *R*_*i*_ (*i* = 0, 2), corresponding to rings with no bound peptide (*i* = 0), one bound peptide (*i* = 1) and two bound peptides (*i* = 2) (Figure 2). In some cases, we also observed nonspecific weak binding (or ‘aggregation’) of a third peptide giving rise to the species *R*_3_. This was particularly clear for β_*E.coli*_/**P6** (Figure 2). Quantification of the three- or four-species spectra was performed by considering the free-ring spectra at various charge states *z* as rulers for the recovery of the fractions of the species *R*_0_, *R*_1_, *R*_2_ (and possibly *R*_3_) with the same charge state (Figure 3). This was achieved with a classic linear least-squares method by considering, for each charge state *z,* that:

1 $$ {s}_n\left(m/z\right)={r}_0{s}_0\left(m/z\right)+{\displaystyle \sum_{i=1}^n{r}_i{s}_0\left(m/z-{\mu}_i\right)} $$

where *s*_0_(*m*/*z*) corresponds to the experimental free-ring spectrum and *s*_*n*_(*m*/*z*) to an experimental spectrum showing *n* distinct species at the same charge state (*n* ≤ 3), and *µ*_*i*_ is the shift on *m*/*z* to superimpose *s*_0_(*m*/*z*) onto the peak for *R*_*i*_ in *s*_*n*_(*m*/*z*) (Figure 3). The result obtained by linear regression and producing the best fit with Equation 1 of each experimental composite spectrum is, for each charge state, the set of the fractions *r*_*i*_ for each species *R*_*i*_ (*i* = 0, *n*). A unique set of values *r*_*i*_ was obtained by averaging the values obtained with the spectra of sufficient quality at different charge states. Usually, two consecutive charge states were usable and, for β_*Mycobacterium*_/**P14**, three were exceptionally usable for one experiment. The dispersion of the results obtained at different charge states was small, as shown in Supplementary Figure S5A.

For the determination of *K*_*d*_ values, the resulting small fraction *r*_3_, if any, was simply added to the fraction *r*_2_ since *R*_3_ can only be formed from *R*_2_. With the pair β_*E.coli*_/**P6**, for which *R*_3_ appeared clearly, a specific treatment not merging *r*_3_ with *r*_2_ (see below) allowed us to derive a *K*_*d*_ -like value for the weak binding. Exceptionally, the species *R*_4_ was also detected but its level was very low and it was ignored.

### Determination of the K_d_ Values from MS-ESI Experiments

The two binding sites on each ring are maximally distant from one another since they are located at both ends of a ring diameter (Figure 1) and, therefore, may safely be considered as independent binding sites. As a consequence, in solution, the set of theoretical *r*_*n*_ values only depends on the dissociation constant *K*_*d*_ of the monomer/peptide equilibrium. The equilibrium concentrations *M*_0_ of free monomers, and *M*_1_ of monomers bound to one peptide, are:

2a $$ {M}_0=\frac{1}{2}\left({M}_{tot}-{P}_{tot}-{K}_d+\sqrt{{\left({K}_d+{M}_{tot}+{P}_{tot}\right)}^2-4{M}_{tot}{P}_{tot}}\right) $$

2b $$ {M}_1=\frac{1}{2}\left({M}_{tot}+{P}_{tot}+{K}_d-\sqrt{{\left({K}_d+{M}_{tot}+{P}_{tot}\right)}^2-4{M}_{tot}{P}_{tot}}\right) $$

where *P*_*tot*_ and *M*_*tot*_ are, respectively, the total concentrations of peptide and of protein monomer (twice the β-ring concentration). The corresponding molar fractions are *m*_0_ = *M*_0_ /*M*_*tot*_ and *m*_1_ = *M*_1_ /*M*_*tot*_. The molar fractions *r*_0_, *r*_1_, *r*_2_ of the ring species *R*_0_, *R*_1_, *R*_2_ can then be derived from a thought-experiment consisting in assembling ring dimers by randomly picking monomers among *M*_0_ and *M*_1_ species, which leads to the classical binomial distribution:

3a $$ \begin{array}{ccc}\hfill {r}_0={m}_0^2,\hfill & \hfill {r}_1=2{m}_0{m}_1,\hfill & \hfill {r}_2={m}_1^2\hfill \end{array} $$

To take into consideration peptide loss due to gas-phase dissociation (GPD), Equations 3a have to be modified by introducing the probability *P* of dissociation of ${M}_1$ into ${M}_0.$ The decrease of the fraction of ${M}_1$ and the concomitant increase of that of ${M}_0$ being equal (by definition of *P*) to *P m*_1_, GPD is accounted for by replacing ${m}_0$ by ${m}_0+P\;{m}_1$ and *m*_1_ by $\left(1-P\right){m}_1$ in Equation 3a, which yields:

3b $$ \begin{array}{ccc}\hfill {r}_0={\left({m}_0+P\;{m}_1\right)}^2,\hfill & \hfill {r}_1=2\left({m}_0+P\;{m}_1\right)\left(1-P\right){m}_1,\hfill & \hfill {r}_2={\left(1-P\right)}^2{m}_1^2\hfill \end{array} $$

Equation 3a corresponds to the fractions in solution, whereas Equation 3b corresponds to the fractions actually measured in the spectrometer. Note that *P* is equivalent to the term 1 − *f*_*sat*_ in a study that also focused on taking into account the influence of GPD [13]. Therefore, the fitting of the set of experimental values *r*_0_, *r*_1_, *r*_2_ for various peptide concentrations requires determining *K*_*d*_ and *P*, which was performed by a nonlinear regression procedure. We also introduced the possibility of a third free parameter *α* corresponding to a multiplicative factor for the total peptide concentration. This was done in view of detecting possible errors in the peptide or protein concentrations (or effective concentrations). Indeed, if α is adjusted to a value lower/higher than 1, this means that either the peptide concentration was over/underestimated in comparison with that of the protein or, equally well, that the protein concentration was under/overestimated in comparison with that of the peptide (which alternative is the correct one cannot be decided). Such a method is the rule when using ITC. Each time, it was examined whether using three free parameters (*K*_*d*_, *P* and α) yielded significantly better result than *K*_*d*_, *P* alone with α fixed to 1. When the gain was marginal we reported the results with *K*_*d*_, *P* alone.

Although the weak nonspecific binding most likely arises from aggregation in the gas phase and is not representative of true equilibrium in solution, we examined whether a *K*_*d*_ -like value would account for this weak binding, particularly with β_*E.coli*_/**P6** for which it is clearly visible. For that, the fraction *r*_3_ of beta-ring with three bound peptides was not merged with *r*_2_ and Equations 3b had to be generalized as follows:

4 $$ {r}_0={\left[{m}_0+P\;\left({m}_1+{m}_2\right)\right]}^2,{r}_1=2\left[{m}_0+P\;\left({m}_1+{m}_2\right)\right]\left[\left(1-P\right)\left({m}_1+{m}_2\right)+P\;{m}_{1,2}\right],{r}_2={\left(1-P\right)}^2{\left({m}_1+{m}_2\right)}^2+{P}^2{m}_{1,2}^2+2\left[\left({m}_0+P\;{m}_1+P\;{m}_2\right)\left(1-P\right){m}_{1,2}+{P}^2{m}_{1,2}^2\right]{r}_3=2\left(1-P\right){m}_{1,2}\left[\left(1-P\right)\left({m}_1+{m}_2\right)+P\;{m}_{1,2}\right], $$

with *m*_2_ standing for the molar fraction of monomers with only one weakly bound peptide and *m*_1,2_ for the molar fraction of doubly occupied monomers (both in the strong specific and weak nonspecific binding sites). Making *m*_2_ = *m*_1,2_ = 0 in Equations 4 yields Equations 3b and *r*_3_ = 0, as expected. The very small fraction *r*_4_ of beta-rings with four bound peptides was ignored. The variations of the molar fractions *r*_0_, *r*_1_, *r*_2_ , *r*_3_ with the concentration *P*_*tot*_ were then fit with a simple model of two independent sites with dissociation constants *K*_*d*1_ ≈ *K*_*d*_ and *K*_*d*2_ > > *K*_*d*_. This obviously does not mean that the weak interaction is mediated by a single site, as the unique *K*_*d*2_ may well lump together the influence of several weak aggregation sites (possibly following a Poisson distribution as explained in [21]). Note also that using a single value *P* to represent the GPD for both sites, instead of *P*_1_ with *K*_*d*1_ and *P*_2_ with *K*_*d*2_, is a simplification justified *a priori* by the need of minimizing the number of free parameters and *a posteriori* by the fact that no correlation between *K*_*d*_ and *P* was observed (Supplementary Figure S7).

### ITC Measurements and ITC-Data Processing

We used an iTC200 from Microcal (now Microcal-Malvern, UK) operated in the high-gain mode. The experimental procedure was described in [16]. In short, the *β* -rings were initially in the cell at concentrations ranging from 20 to 60 μM and the peptides were in the syringe at concentrations ranging from 200 to 600 μM. The buffer was Hepes 10 mM, pH 7.4, supplemented with NaCl 150 mM and EDTA 3 mM. Experiments were performed at different temperatures from 15 to 34 °C.

The processing of the ITC data was first performed with the same model as the one used for ESI-MS (one specific binding site per monomer). Since the titration experiments with the same protein-peptide pair were performed at different temperatures, we used a procedure that we have termed ‘Global Thermodynamic Treatment’ (GTT), wherein all titration experiments at different temperatures have to be fit at once, and not separately [22]. The advantage of this still unusual method is of fitting several titration curves with a minimum set of free parameters. All *K*_*d*_ values obtained by ITC were corrected for 22.5 °C, which was the incubation temperature used in ESI-MS experiments. This was performed by using the Van’t Hoff equation: ∂ ln *K*_*d*_/∂*T* = − *ΔH*/(*RT* ^2^), where *ΔH* is the variation of enthalpy during complex formation and ***R*** = 8.314 J mol^−1^ K^−1^ the gas constant. Note that such a correction is quite justified for *K*_*d*_ values obtained by ITC since the primary output from ITC is precisely the enthalpic term *ΔH* governing the evolution of *K*_*d*_ with the temperature. In addition, the extrapolation of *K*_*d*_ at 22.5 °C was always performed from a temperature close to 22.5 °C.

The latter simple processing method did not allow us to obtain a correct fit of several ITC data and, therefore, we attempted to use a more complex (but classic) model by considering a competition between two modes of binding on the same site. Note that the results from ESI-MS imposed to use a competition model for the same site, and not the binding to another site, since ESI-MS did not show the binding of a second peptide per monomer with sufficient affinity to explain the observed lacks of fit. This competition model requires determining a fixed fraction *f*_1_ of the protein monomers binding their ligand according to a dissociation constant *K*_*d*1_ and an enthalpy variation *ΔH*_1_, and a fixed fraction *f*_2_ = 1 − *f*_1_ binding their ligand according to a dissociation constant *K*_*d*2_ and an enthalpy variation *ΔH*_2_ (we consider *K*_*d*2_ < *K*_*d*1_).

Finally, since it appeared clearly with the ESI-MS data from β_*E.coli*_/**P6** (Figure 2) that a third peptide (and marginally a fourth peptide) could bind the *β* -ring, it was attempted to see whether this weak association was detectable in the ITC data from β_*E.coli*_/**P6**. At variance with the previous situation, it was thus necessary to use a binding model with (at least) two independent binding sites on each monomer to account for the simultaneous binding of three peptide molecules per dimer.

All data processing was performed with our own methods programmed with *Mathematica* (Wolfram Research).

## Results and Discussion

### Determination of the Fractions *r*_*n*_ by Fitting of the Experimental Spectra

**Figure 4:**
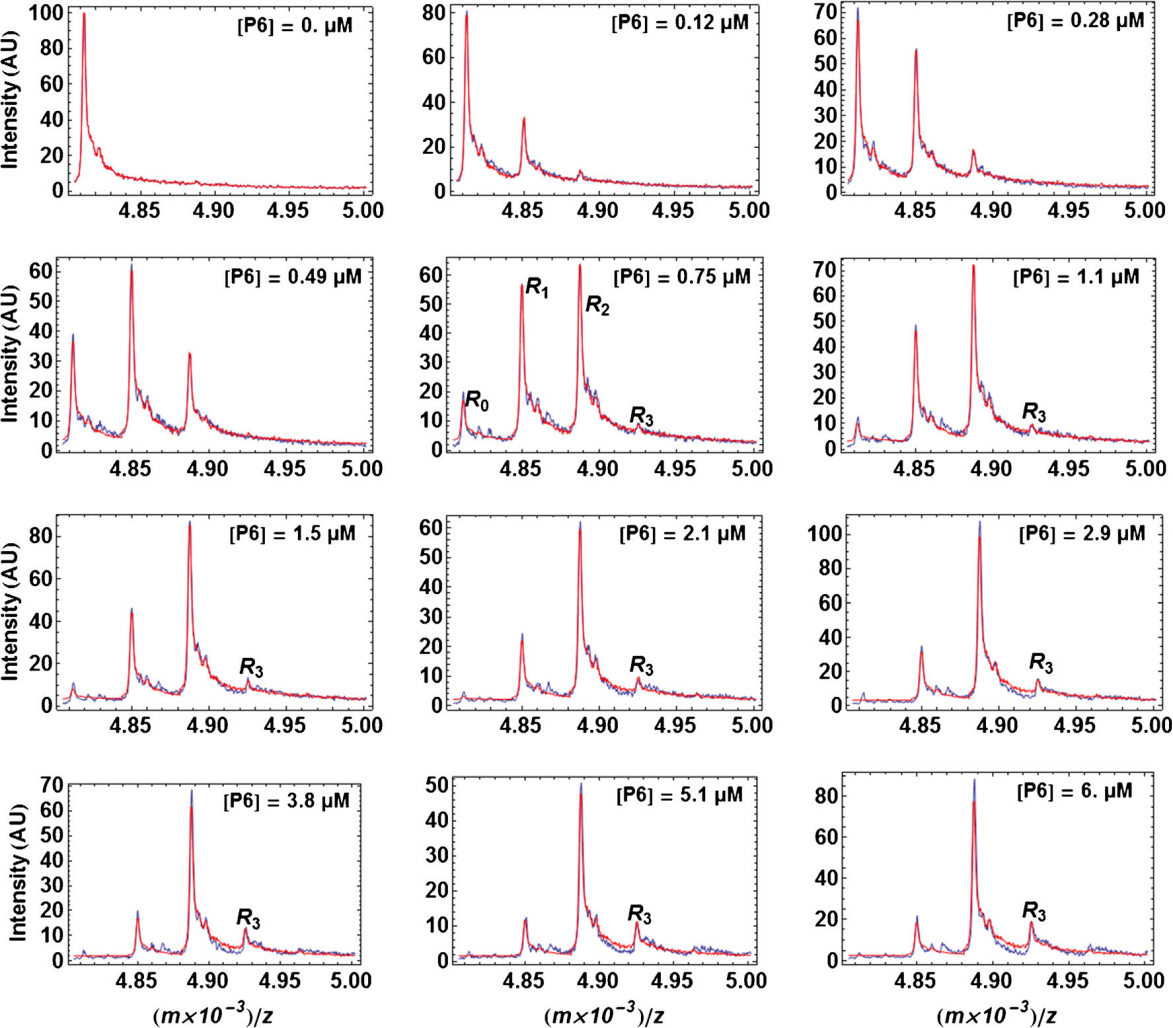
Fit of the experimental spectra for β_*E.coli*_ */*P6. The experimental spectra for z = +18 (blue curves) are those in Figure 2 for β_*E.coli*_/**P6** and, for three of them (0, 0.28, and 2.9 μM), correspond to the spectra in Figure 3 (with z = +17, hence the differences in *m/z*). The theoretical spectra (red curves) obtained from Equation 1 are also shown. The peak for *R*_3_ at *m/z* ≈ 4925 starts emerging at [P6] = 0.75 μM and then appears clearly. A very faint fourth peak corresponding to the species *R*_4_ begins to appear at the highest concentrations of **P6** but was ignored

The raw experimental spectra are in Figure 2 and Supplementary Figure S3. Although the individual peaks are broad, they show well the successive apparition of *R*_1_, *R*_2_ (and possibly *R*_3_) species upon peptide concentration increase. Figure 4 (β_*Ecoli*_ /**P6**) and Supplementary Figure S4A (β_*Pseudomonas*_/**P14**) show two examples of fits obtained for the complete experimental spectra by using the free-ring spectrum as a ruler. For these two cases, the results were very good (particularly for β_*Pseudomonas*_/**P14** in Supplementary Figure S4A) due to the shape of each free-ring spectrum being quite representative of the shape of each peak in the composite spectra. However, this was not always the case and, in situations where there was a significant broadening of all peaks (obtained in presence of peptide) in comparison of the peak *R*_0_ serving as a ruler (obtained without peptide), the quality was affected, as expected. We can only invoke for this dependence of spectral quality on the nature of the beta ring and on the nature of the peptide a variable influence of desalting and of DMSO. The worst result was for β_*Pseudomonas*_/**P11** shown in Supplementary Figure S4B. One may consider for the future an improvement of the fitting method by constructing, when necessary, a composite ruler. Indeed, Supplementary Figure S4B shows that overall the peaks retain the same composite shape during a titration. In fact, this method could be put into practice simply by using as a ruler the peak *R*_0_ obtained at the lowest ligand concentration, not at null concentration.

**Figure 5:**
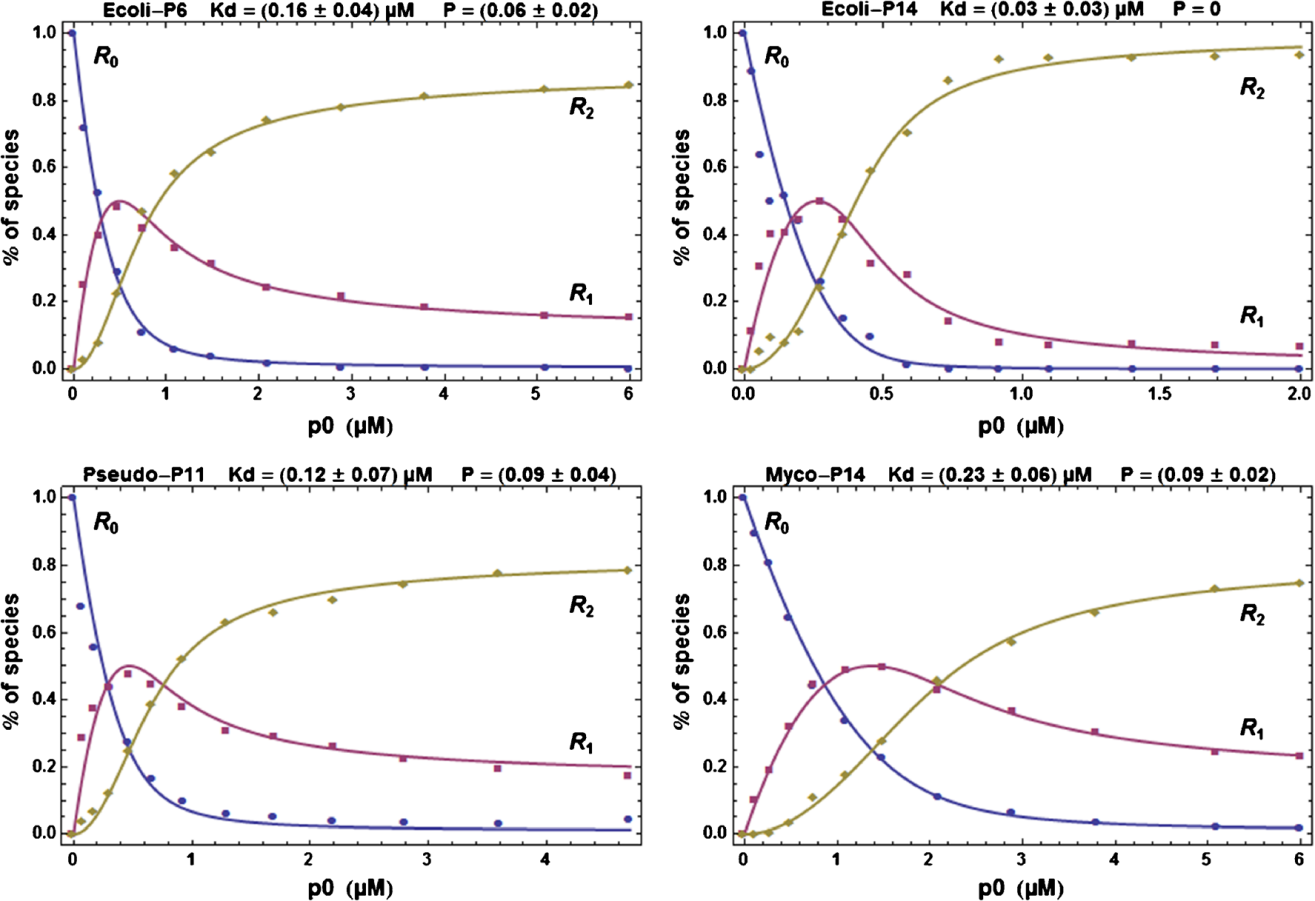
Experimental fractions of the species *R*_0_, *R*_1_, *R*_2_ and theoretical curves. Experimental fractions for the species *R*_0_, *R*_1_, *R*_2_ and theoretical fits (solid curves) with the indicated values for *K*_*d*_ and *P* for a subset of all titration curves. All other curves are in Supplementary Figure S5B. The analysis of the errors and the correlation between parameters are shown in Supplementary Figure S6. The inclusion of *R*_3_ in the processing is described later in the text for β_*Ecoli*_ /**P6** and the result is shown in Supplementary Figure S5C

From these fits, we obtained the sets of the fractions *r*_*n*_ of all *R*_*n*_ species for each ligand concentration. A subset of the results is shown in Figure 5 and all results in Supplementary Figure S5B. In all situations, the evolutions of the concentrations of the three species *R*_0_, *R*_1_, *R*_2_ are well-defined with a maximum for the intermediate species *R*_1_. It is also to be noted that there is a clear lag in the appearance of *R*_2_ (see, in particular, β_*Mycobacterium*_/**P14** in Figure 5), which is in line with the expectation since *R*_2_ can appear only after *R*_1_ has sufficiently accumulated.

### Determination of *K*_*d*_ and GPD Probability *P* Values

These experimentally-derived titration curves in Figure 5 were fit by using Equations 2a, 2b and Equation 3b to obtain the values of *K*_*d*_, *P* (and possibly α). Figure 5 also shows the best fit obtained after minimization of the residual sum of squares (SOS). An analysis of the parameter errors and correlations shows systematically the same pattern (Supplementary Figure S6). In all situations, the SOS has a well-defined minimum and the pairs *K*_*d*_ and *P*, or *K*_*d*_ and α are anti-correlated in such a way that increasing/decreasing *K*_*d*_ slightly from its best value minimizing the SOS is compensated for by decreasing/increasing slightly *P* or α from their best values.

The probabilities *P* of GPD that we obtained never exceed 12%, which is in line with the low cone voltage fixed to 100 V. Such a maximum value of 12% is low in comparison with the values reported in [13] that ranged from 0 to 75%. However, in [10]*,* the cone voltage was tuned differently for each ligand and it is likely that values higher than 100 V were used. We note that the distribution of these probability values does not show any correlation with the affinity of the peptide ligand for its protein target but, instead, shows a clear correlation with the nature of the peptide (Supplementary Figure S7). This is in agreement with the conclusion from another study on protein–peptide interactions [23]. Gathering the results from [13] (which, apart one case among six, did not bear on protein–peptide interaction) also revealed a complete lack of correlation between ${K}_d\left(\mathrm{E}\mathrm{S}\mathrm{I}\hbox{--} \mathrm{M}\mathrm{S}\right)$ and *P* (not shown). This should not come as a surprise since a *K*_*d*_ value results from the influence of both the association and dissociation steps, whereas the association step cannot have any influence in the gas phase essentially devoid of ligand. Therefore, only the dissociation step influences the value of *P* and, indeed, GPD is correlated with the activation energy for dissociation [4].

### Misleading Results from ITC

**Figure 6:**
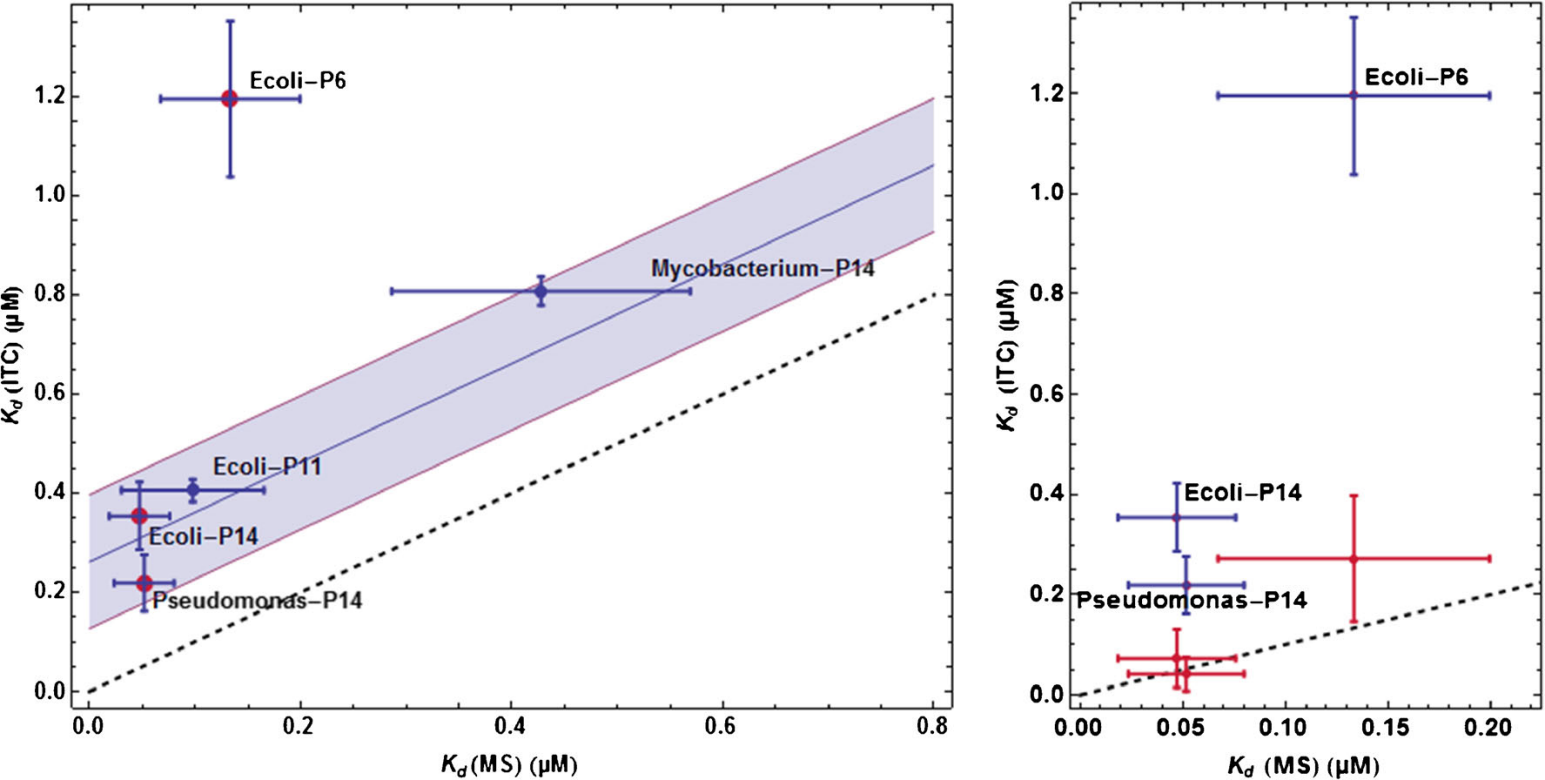
Comparison of Kd(MS-ESI) and Kd(ITC) results. **Left panel:** processing of ITC data with one mode of binding. Each point is the average of all separate experiments for a given protein-peptide pair. The dashed line represents K_d_(ITC) = K_d_(MS-ESI). In one thus obtained K_d_(MS-ESI) < K_d_(ITC) in all situations and, apart for the clear outlier β_*E.coli*_/**P6**, it appears that K_d_(ITC) – K_d_(MS-ESI) ≈ 0.25 μM. The shaded strip of width 0.3 μM represents the 95% confidence interval for K_d_(ITC) – K_d_(MS-ESI), which means that excluding β_*E.coli*_/**P6**, one has the inequality 0.15 μM < K_d_(ITC) – K_d_(MS-ESI) < 0.35 μM with a probability of 95%. The data highlighted with a red dot are re-examined in the right panel. **Right panel:** processing of ITC data with two modes of binding on the same site. The ITC data for β_*E.coli*_/**P6**, β_*E.coli*_/**P14** and β_*Pseudomonas*_/**P14** (red dots in the left panel) could be processed with a model involving two modes of binding on the same specific site (see text). This led to a high- and a low-affinity binding. The dark-blue symbols correspond to the previous results in the left panel and the red symbols correspond to the new ITC results for the high-affinity mode of binding (the ITC results for the low-affinity mode of binding are close to the results in the left panel and are shown in Supplementary Figures S9 and S10). In all three cases these high-affinity K_d_(ITC) values are close to the dashed line representing K_d_(ITC) = K_d_(MS-ESI)

The ITC titration curves and their fits with the same model as for ESI-MS data processing are shown in Supplementary Figure S8. The comparison of the ${K}_d\left(\mathrm{E}\mathrm{S}\mathrm{I}\hbox{--} \mathrm{M}\mathrm{S}\right)$ and available *K*_*d*_(ITC) values is shown in Figure 6 (left panel). It appears that apart for an obvious outlier (β_*E.coli*_/**P6**), the two sets of results show good correlation. However, the ${K}_d\left(\mathrm{E}\mathrm{S}\mathrm{I}\hbox{--} \mathrm{M}\mathrm{S}\right)$ values were systematically lower than those from ITC. Letting aside β_*E.coli*_/**P6**, this inequality may be represented by ${K}_d\left(\mathrm{I}\mathrm{T}\mathrm{C}\right)\approx {K}_d\left(\mathrm{E}\mathrm{S}\mathrm{I}\hbox{--} \mathrm{M}\mathrm{S}\right)+0.25\;\mu \mathrm{M}$. This is only intended to represent in as simple a way as possible the relationship between the two sets of results.

It is often mentioned that an increase of the ligand concentration during the desolvation step might induce an apparent increase in the affinity, and thus a ${K}_d\left(\mathrm{E}\mathrm{S}\mathrm{I}\hbox{--} \mathrm{M}\mathrm{S}\right)$ lower than *K*_*d*_(ITC) in solution, as we observed. However, we note that this tendency ${K}_d\left(\mathrm{I}\mathrm{T}\mathrm{C}\right)>{K}_d\left(\mathrm{E}\mathrm{S}\mathrm{I}\hbox{--} \mathrm{M}\mathrm{S}\right)$ was not apparent in the results from [13]. Furthermore, as explained in the ‘Experimental section,’ the model in use was too simple and a more sophisticated processing of the ITC data with two competing binding modes on the same site revealed another *K*_*d*_(ITC) very close to ${K}_d\left(\mathrm{E}\mathrm{S}\mathrm{I}\hbox{--} \mathrm{M}\mathrm{S}\right)$ for three peptides (Figure 6, right panel). All fits for the reprocessed ITC curves are in Supplementary Figure S9 and all resulting parameter values are in Supplementary Figure S10.

A striking conclusion arises from inspection of the results. Recall first that processing the ITC curves with the simplest usual model implies introducing a single pair of thermodynamic parameters (*K*_*d*_, *ΔH*). On one hand, several fits were of insufficient quality (particularly β_*E.coli*_/**P14** at 20 °C, Supplementary Figure S8). On the other hand, in several occasions the fits would have been accepted as such in most routine ITC studies (e.g., β_*E.coli*_/**P6** at 26 and 30 °C, β_*E.coli*_/**P14** at 30 °C in Supplementary Figure S8). Since we know that lower *K*_*d*_ values than those obtained by ITC (with the simplest model) are necessary to explain the ESI-MS titration curves, it is important to examine what kind of error is made if one remains content with the ITC results obtained with the simplest model. From Supplementary Figure S10 it appears that the model with two binding modes led to the ‘blue’ pair (*K*_*d*1_, *ΔH*_1_) and ‘red’ pair (*K*_*d*2_, *ΔH*_2_). For example, with β_*E.coli*_/**P6**, one obtained (*K*_*d*1_ ≈ 1 μM, *ΔH*_1_ ≈ 1 kcal mol^−1^) and (*K*_*d*2_ ≈ 0.25 μM, *ΔH*_2_ ≈ –12 kcal mol^−1^). Importantly, the simple method involving one binding mode led (at 25 °C) to the unique pair (*K*_*d*_ = 1.4 ± 0.2 μM, *ΔH* ≈ –11.8 ± 0.2 kcal mol^−1^), which is striking as this corresponds to mixing of the two previous pairs by reporting the *K*_*d*_ value of the weaker binding mode, but the *ΔH* value of the stronger binding mode. The reason for which this was possible stems from the very low |*ΔH*| value of the weaker binding mode, which almost erased its contribution in the overall amplitude of the titration curves, whereas the higher *K*_*d*_ value (lower affinity) attached to this low |*ΔH*| value did influence the overall sigmoid shape of the titration curve by making it not too sharp. As a consequence, the too simple model ‘did its best’ to explain both the significant amplitude of the titration curves and the not-too-sharp return to base line by mixing wrongly the parameters from the two binding modes. Examination of Supplementary Figure S10 shows that the same problem arose in all other situations. To our knowledge, this is the first time that this kind of serious error is identified and established clearly thanks to ESI-MS.

### Determination of a *K*_*d*_ for Describing Weak Nonspecific Binding (Aggregation)

Supplementary Figure S5C (left panel) shows how the processing of the raw MS-ESI data from β_*E.coli*_/**P6** (Figure 2) led to the determination of the fraction *r*_3_ besides those for *r*_0_, *r*_1_, *r*_2_. Fitting these data with Equations 4 led to convincing results with a value *K*_*d*1_ = (0.166 ± 0.04) µM for the specific site very close to *K*_*d*_ = (0.16 ± 0.04) µM obtained by merging the fraction *r*_3_ with *r*_2_ (Supplementary Figure S5C, right panel), whereas *K*_*d*2_ for the nonspecific weak site converged to the much higher value *K*_*d*2_ = (42.6 ± 3) µM. The errors on *K*_*d*1_ and *K*_*d*2_ were estimated roughly by comparison with the results obtained from a duplicate experiment. It thus appears that both the specific and nonspecific bindings were very well characterized.

Importantly, all attempts at processing the ITC data from β_*E.coli*_/**P6** with the same binding model as the one used for MS-ESI, first, did not improve significantly the fit of the experimental titration curves shown in Supplementary Figure S8 and, second, led to extremely unstable results upon minor changes in baseline determination. For example, the value for *K*_*d*2_ could jump from a reasonable value in the range of 20–30 μM (as observed with MS-ESI) to a value in the range 10–30 mM. One has thus to admit that no convincing results could be obtained by ITC with a binding model requiring two (or more) binding sites per monomer, which supports well that the nonspecific weak binding resulting from ‘aggregation’ due to the particular conditions in the ESI-MS experiments.

Although the peak for the species *R*_3_ for β_*Mycobacterium*_/**P14** is visible on the raw spectra (Figure 2), the quality of the data was not sufficient for repeating successfully the same kind of analysis. For the complexes β_*E.coli*_/**P11** and β_*Pseudomonas*_/**P11**, the signal for the species *R*_3_ was too faint (Supplementary Figure S3) to attempt any processing. For the complexes β_*E.coli*_/**P14** and β_*Pseudomonas*_/**P14**, no signal for the species *R*_3_ was detected. However, **P14** has the highest affinity for *E. coli* and *Pseudomonas* β-rings and, for this reason, the ESI-MS titrations did not use a concentration above 2 μM for **P14**, whereas the peptide concentration reached 4.7 and 6 μM for the other peptides (Table 1). This most likely explains why weak nonspecific binding was not observed with β_*E.coli*_/**P14** and β_*Pseudomonas*_/**P14**.

### Experimental Factors Limiting the Accuracy of MS-ESI Processing

At the basis of the method in use is the fit of the experimental spectra (Figure 4) with the method described in Figure 3 to obtain, at each ligand concentration, the set of the fractions *r*_*n*_ for each *R*_*n*_ species. It was rather surprising that obtaining an excellent fit of the experimental spectra was not sufficient to obtain a titration curve of excellent quality with the experimental points being smoothly distributed as with β_*Mycobacterium*_/**P14** in Figure 5. Only a set of such smooth curves may lead to a very low residual sum of squares (SOS), whereas a more erratic distribution of the experimental points (as with β_*E.coli*_/**P14** in Figure 5), being the mark of significant experimental noise, cannot lead to a very low SOS. Upon examination of the distribution of all SOS values, it appeared that these are anti-correlated with the maximum value of the peptide concentration used in the experiment (Supplementary Figure S11). The interpretation of this observation is clear: when the affinity of the protein–peptide interaction was high, the maximum peptide concentration in use for the titration did not exceed 2 μM, which led to smaller dispensed volumes of the peptide stock solutions in comparison with situations where the maximum peptide concentration reached 6 μM. As a consequence, a lower relative accuracy of the final peptide concentration was obtained for the titrations performed with a maximum peptide concentration of 2 μM, which explains well the observed tendency in Supplementary Figure S11. Examination of Figure 6 (left panel), however, shows that this lower relative accuracy cannot account for the observed inequality ${K}_d\left(\mathrm{I}\mathrm{T}\mathrm{C}\right)>{K}_d\left(\mathrm{E}\mathrm{S}\mathrm{I}\hbox{--} \mathrm{M}\mathrm{S}\right)$ for the low *K*_*d*_ values.

## Conclusion

We have obtained the *K*_*d*_ values for the binding of peptides onto DNA-processivity β-rings from three bacterial organisms. Our ESI-MS data show very well the successive specific binding of two peptides per ring and, in several occasions, the nonspecific binding of a third peptide. We obtained accurate titration curves that often could only be processed to obtain the ${K}_d\left(\mathrm{E}\mathrm{S}\mathrm{I}\hbox{--} \mathrm{M}\mathrm{S}\right)$ values by taking into account the possibility of peptide loss due to GPD. In comparison, the fit of the ITC curves with the same model of binding was often poor and led to *K*_*d*_(ITC) values linked to their ESI-MS counterparts by ${K}_d\left(\mathrm{I}\mathrm{T}\mathrm{C}\right)\approx {K}_d\left(\mathrm{E}\mathrm{S}\mathrm{I}\hbox{--} \mathrm{M}\mathrm{S}\right)+0.25\;\mu \mathrm{M}$. A more sophisticated model involving two modes of binding on the same site improved greatly the results by obtaining in three cases an additional *K*_*d*_(ITC) value compared well with ${K}_d\left(\mathrm{E}\mathrm{S}\mathrm{I}\hbox{--} \mathrm{M}\mathrm{S}\right)$. Our results highlight the complementarity of the two techniques in use but also how ESI-MS can help obtain the correct results with ITC in ambiguous situations. On one hand, ESI-MS did not detect the weaker binding mode with the experimental procedure in use. In fact, it would be necessary to repeat these ESI-MS experiments by considering higher concentrations of the peptides and of the proteins to check whether this weaker binding mode is really present in the MS conditions. One could increase the protein concentration 10-fold (i.e., up to 6 μM), but certainly not up to the concentration range used in the ITC experiments (30–60 μM), which would lead to saturation of the instrument response and loss of linearity between concentration and peak amplitude. On the other hand, it happened that ITC detected with difficulty the major and stronger binding mode. In usual situations involving routine application of ITC, one may even consider that such ITC data would lead to serious errors by reporting the *K*_*d*_ of the weaker and minor binding mode and the *ΔH* of the stronger and major binding mode. It is likely that such errors are to be found in published data.

One potential problem that may have plagued ITC measurements (and would explain why the weaker binding mode was absent in ESI-MS conditions), comes from the limited solubility of the peptides used. It is to be recalled that the peptides were in the syringe at a concentration ten times higher than the initial concentration of the protein in the cell. These peptide concentrations ranged from 200 to 400 μM for β_*E.coli*_/**P14** and β_*Pseudomonas*_/**P14**, but reached 500 μM for β_*Mycobacterium*_/**P14** and even 600 μM for β_*E.coli*_/**P6**. These are high concentrations and, although no problem was apparent during the ITC experiments, it is likely that the activity of the peptides was significantly less than for an ideal solution at these concentrations and, as a consequence, the affinity of these peptides for their target was apparently lower. In particular, it may be noted that β_*E.coli*_/**P6**, for which the discrepancy between MS-ESI and ITC was the highest (Figure 6), corresponds precisely to the highest peptide concentration used in the ITC experiments. In comparison, the concentrations used in ESI-MS titrations were much smaller (Table 1). Furthermore, DMSO was used systematically in the ESI-MS experiments to prevent aggregation, whereas this was avoided in the ITC experiments because any discrepancy in the DMSO concentrations in the syringe and in the measurement cell is a common source of parasitic heat signal (heat of dilution). A possible explanation for the observed discrepancy between the two techniques is thus linked to this solubility problem and to segregation of the peptides in the ITC experiments into a solubilized fraction (probably *ca.* 80% according to the values of *f*_1_ in Supplementary Figure S10) and a more or less aggregated fraction (*ca.* 20%) that would be unable to interact with the protein. This is a conclusion of broad interest given the increasing importance of ITC for the characterization of molecular interactions. Clearly, additional investigations are needed for a better description of such situations. In particular, one might consider that the ITC model with two modes of binding could merely be an efficient numerical description of ‘abnormal’ ITC titration curves and that a more sensible model should take into account how each peptide is solubilized, which might require taking into account more or less slow kinetics.

One major lesson from this work is that a homodimer having two identical and independent binding sites is very favorable for deriving the *K*_*d*_ and the probability *P* of GPD by ESI-MS experiments. We are confident that this should hold true for any situation neither involving too low affinity nor too important GPD; to which extent remains to be evaluated. In the general situation where the favorable feature of a homodimer does not exist, one may imagine to obtain dimers artificially, for example by tandem expression of two monomers separated by a short flexible linker. Obviously, one cannot expect this to be a common practical solution due to the additional necessary work and to possible folding problems. However, one may hope that the technical possibilities of linking protein monomers (for example by specific N- or C-term ‘adhesive’ tags) will expand sufficiently to transform ESI-MS into a competitive routine method for *K*_*d*_ determination. Various lines of investigation currently pursued by several laboratories add weight to this suggestion [24–26]. Of course, only ITC allows measuring directly both the *K*_*d*_ and the *ΔH*, and thus only ITC allows to obtain the complete thermodynamic signature of an interaction; however, in view of the problems described in this work, using both methods in parallel may well be the best way of avoiding wrong interpretations in difficult situations. In particular, only ESI-MS can tell unambiguously whether a non-simple binding mechanism requires considering either two binding modes competing for the same site, or the binding on different sites.

## Electronic supplementary material

Below is the link to the electronic supplementary material.

ESM 1 (DOCX 4747 kb)
