# Supplementary material for: Native ESI Mass Spectrometry Can Help to Avoid Wrong Interpretations from Isothermal Titration Calorimetry in Difficult Situations
Source: J Am Soc Mass Spectrom. 2016 Dec 12;28(2):347–57. doi: 10.1007/s13361-016-1534-6 (PMC5227004; doi:10.1007/s13361-016-1534-6)
Supplement: Supplementary file 1 — (DOCX 4747 kb) [file 13361_2016_1534_MOESM1_ESM.docx]

**Supplementary Materials for**

**Native ESI mass spectrometry can help to avoid wrong interpretations from isothermal titration calorimetry in difficult situations**

Philippe Wolff^‡^, Cyrielle Da Veiga^‡^, Eric Ennifar^‡^,  Guillaume Bec^‡^, Gilles Guichard^¥^, Dominique Burnouf^‡^* & Philippe Dumas^‡¶^*

^‡^Biophysics & Structural Biology team

Unité Architecture et réactivité de l’ARN (UPR9002)

Institut de Biologie Moléculaire et Cellulaire du CNRS

Université de Strasbourg

15, rue René Descartes F67084 Strasbourg cedex, France

^¶^ Institut de Biologie et Génétique Moléculaire

Université de Strasbourg

1, rue Laurent Fries, 67400 Illkirch

^¥^ (1) Université de Bordeaux, CBMN, UMR 5248, Institut Européen de Chimie et Biologie, 2 rue Robert Escarpit, 33607 Pessac, France.

(2) CNRS, CBMN, UMR 5248, 33600, Pessac, France

*Corresponding authors

p.dumas@ibmc-cnrs.unistra.fr [p.dumas@igbmc.fr](mailto:p.dumas@igbmc.fr)

[d.burnouf@ibmc-cnrs.unistra.fr](mailto:d.burnouf@ibmc-cnrs.unistra.fr)

**To which extent can a singleexplain ESI-MS data affected by Gas Phase Dissociation?**

We consider here a monomeric macromolecule with a single binding site. The notations are the same as in the main text with $P_{tot}$and$M_{tot}$being the total ligand and macromolecule concentrations, respectively. (To avoid multiple notations,$P_{tot}$ is used here with the general meaning of ligand concentration rather than merely peptide concentration since the present analysis is general). From equations (2a,b), it can be obtained (all following calculations were done with *Mathematica*):

$\frac{m_{1}}{m_{0}}=\frac{P_{tot}-M_{tot}-K_{d}+\left[\left(P_{tot}+M_{tot}+K_{d}\right)^{2}-4P_{tot}M_{tot}\right]^{1/2}}{2 K_{d}}$ (S1)

The decrease of the fraction $m_{1}$ of$M_{1}$and the concomitant increase of the fraction $m_{0}$of$M_{0}$being equal (by definition of $P$) to $P m_{1},$ GPD is accounted for by replacing $m_{0}$by $m_{0}+P m_{1}$and $m_{1}$ by $\left(1-P\right)m_{1}$, which means that the ratio$m_{1}/ m_{0}$valid in solution becomes $\left(1-P\right)m_{1}/\left(m_{0}+P m_{1}\right)$after introduction in the spectrometer. Analogously, from equations (2a,b), it can be obtained:

$\frac{ \left(1-P\right)m_{1}}{ m_{0}+P m_{1} }=\frac{2\left(P-1\right)P_{tot}}{K_{d}+M_{tot}+\left(2P-1\right)P_{tot}+\left[\left(P_{tot}+M_{tot}+K_{d}\right)^{2}-4P_{tot}M_{tot}\right]^{1/2}}$ (S2)

Now, if we ignore that GPD has occurred, we will consider the latter ratio as equal to $m_{1}/ m_{0}$ from equation (S1), which will lead to the determination of an apparent $K_{d}^{app}$ instead of the real$K_{d}$. Solving for $K_{d}^{app}$to obtain identical values of the two ratios from equations (S1) and (S2) leads to:

$\frac{ K_{d}^{app}}{ K_{d} }=\frac{P\left\{\left(P-2\right)\left[\left(p_{tot}+m_{tot}+1\right)^{2}-4p_{tot}m_{tot}\right]^{1/2}-\left(p_{tot}+m_{tot}\right)P\right\}-P\left(P-2\right)-2}{2\left(P-1\right)}$ (S3)

where we introduced the dimensionless concentrations$p_{tot}=P_{tot}/K_{d}$and $m_{tot}=M_{tot}/K_{d}$. This shows to which extent the ratio $K_{d}^{app}/K_{d}$is variable when$p_{tot}$and $m_{tot}$are varied during a titration experiment. Obviously, if $K_{d}^{app}/K_{d}$varies little, $K_{d}^{app}$may be seen as constant (possibly, by invoking unduly measurement errors) and thus as the correct answer; in such a situation, it becomes difficult to discriminate a situation with GPD ($P\neq0$) from a situation without GPD ($P=0$). Equation (S3) is represented in Fig. S1 with parameters corresponding to different situations. It is seen that, in most cases, there is effectively little variation of $K_{d}^{app}/K_{d}$ (graphs with red labels), apart when $P$has a high value and $m_{tot}=M_{tot}/K_{d}$a low value (graphs with black labels). In the worse situation corresponding to $P =0.1$and$m_{tot}=5$, a well-defined value $K_{d}^{app} \approx\left(1.7\pm0.1\right) K_{d}$would explain perfectly the experimental data even though GPD has been ignored. Admittedly, an error on $K_{d}$ by a factor of 1.7 would certainly be considered as a minor one, but with $P =0.4$and$m_{tot}=5$, $K_{d}^{app} \approx(6\pm1) K_{d}$would also explain reasonably well the experimental data, but now at the expense of an unacceptable level of error, without mentioning $P =0.7$and$m_{tot}=5$ leading to an error by a factor of the order of 20. Importantly, comparing the two rightmost graphs shows that, using a low value of$m_{tot}=M_{tot}/K_{d}$(0.2 instead of 5), with the same high level of GPD $\left(P =0.7 \right)$would clearly reveal that a single value of $K_{d}^{app}$is fully unacceptable (25-fold variation of $K_{d}^{app}$ to be compared with a 1.5-fold variation). This conclusion that, everything else being the same, a low value of$M_{tot}/K_{d}$is more discriminative was already reached in [1]. However, there is obviously a limit for very low $K_{d}$ implying too low concentrations of the macromolecule.

**
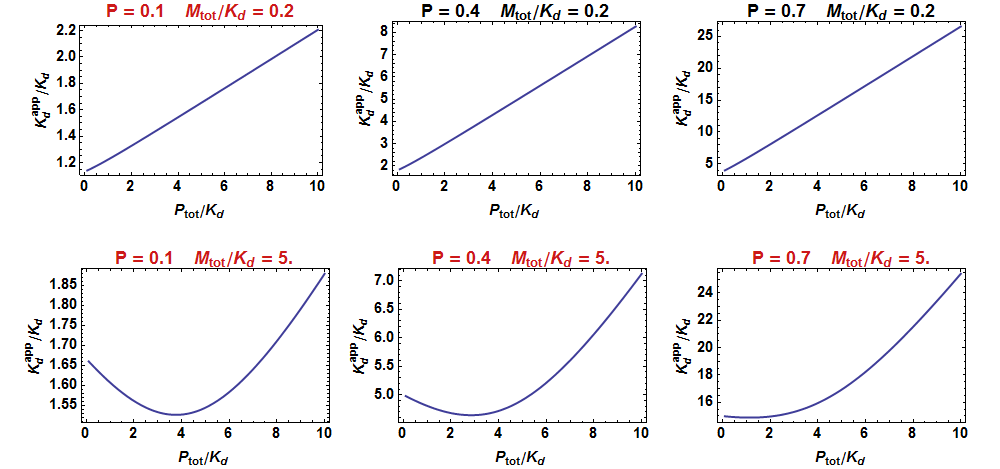
**

**Fig. S1 Analysis of the effect of GPD on the determination of** $K_{d}$.

The graphs with a red label correspond to situations where a single$K_{d}^{app}$ would explain ‘reasonably well’ the data from a titration affected by GPD without taking GPD in consideration during data processing. By ‘reasonably well’, it is meant by considering that the data are affected by experimental errors and that too small variations of$K_{d}^{app}$would not be considered as relevant. Here, a red label is used when the ratio $K_{d}^{app} /K_{d}$does not vary by more than twofold in a usual range of ligand concentration$\left(0<P_{tot} /K_{d} <10\right)$.

**

**

**Fig. S2 Chemical structure formulae of the synthetic peptides P6, P11 and P14.**

**
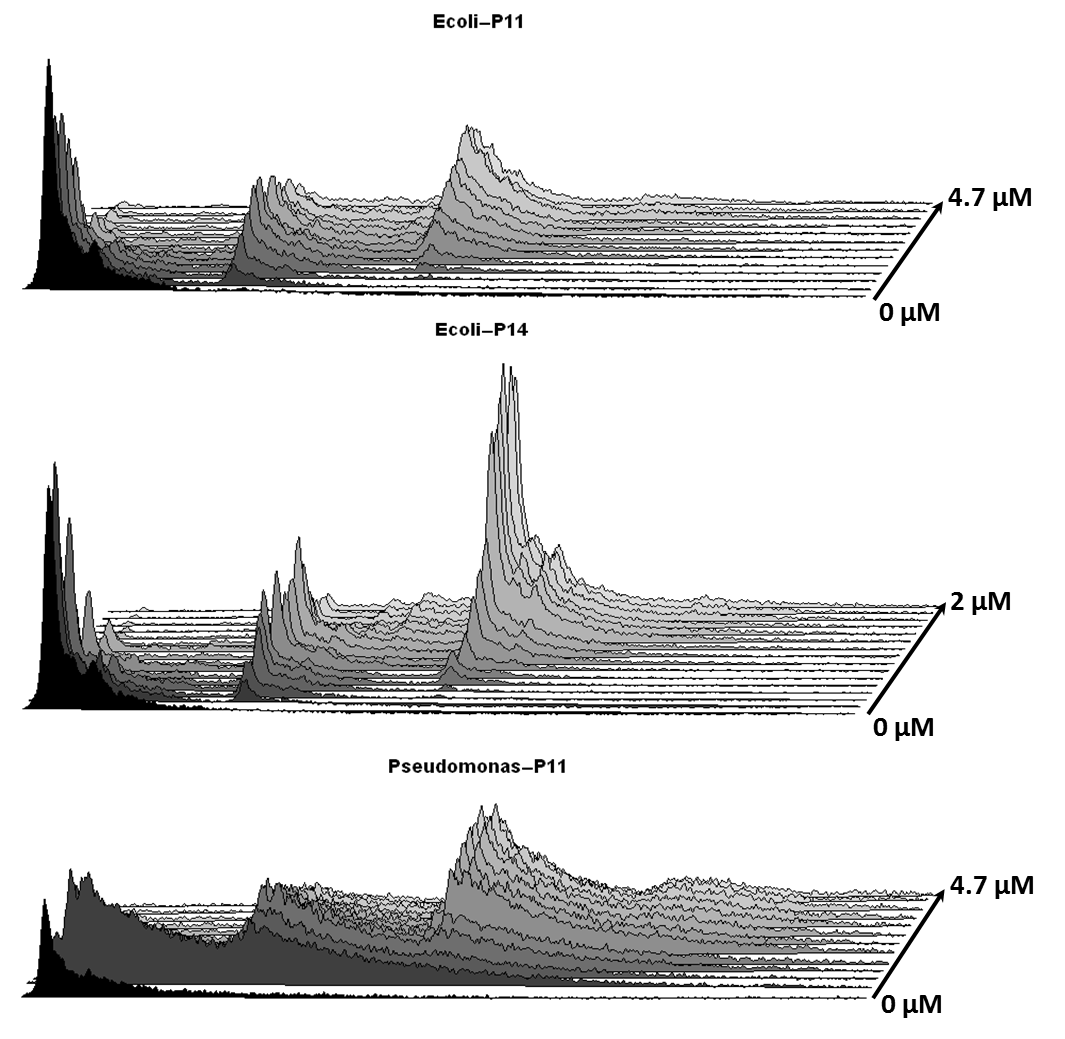
**

**Fig. S3 Raw experimental ESI-MS spectra for the remaining protein-peptide titrations**

**
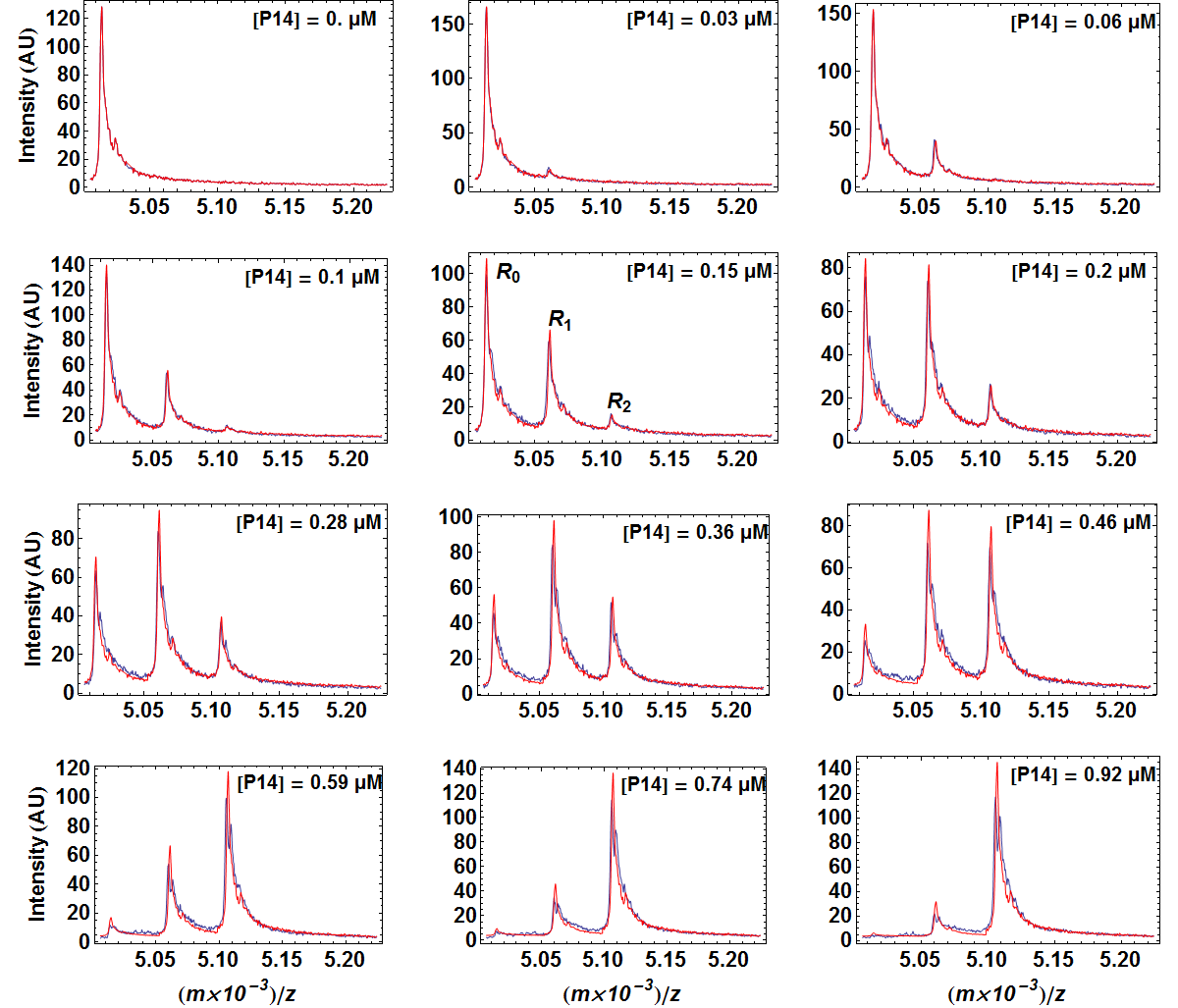
**

**Fig. S4A Fit of the experimental spectra for *Pseudomonas* ring + P14**

The experimental spectra correspond to those shown in Fig. 2 for β*_Pseudomonas_*/**P14**. Here the difference between the experimental and theoretical spectra (red curves) is hardly visible at the scale of this figure, which is the mark of an excellent result. Note the total absence of$R_{3}$ in this case, which may result from the highest peptide concentration ([P14]_max_ = 0.92 µM) being insufficient for populating non-specific binding sites of too low affinity. Compare with β*_E.coli_*/**P6** for which [**P6**]_max_ was 6 µM (Fig. 4).

**
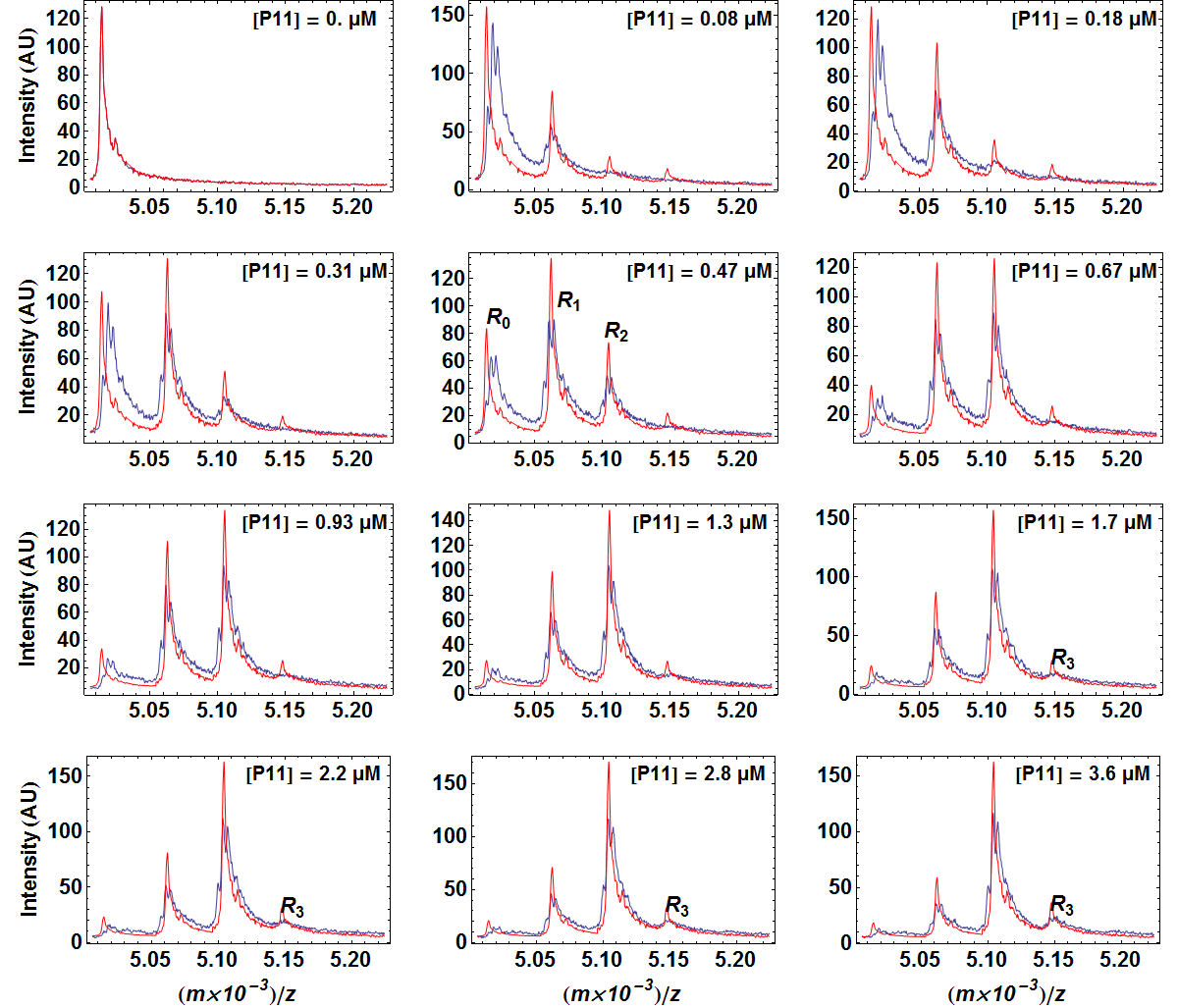
**

**Fig. S4B Fit of the experimental spectra for *Pseudomonas* ring + P11**

This example illustrates the worse fits obtained by using the peak R_0_ without peptide as a ruler. It appears clearly that the problem is due to the broadening of all peaks with peptide in comparison of the peak R_0_ without peptide. The consequences are twofold: the peaks are less sharp than R_0_ (without peptide) and the theoretical peaks in red derived from the latter are too high in comparison of the experimental peaks in blue; this is compensated for by the theoretical tail of each peak being below the experimental tail. See text for a suggestion about a possible improvement of the method in such a situation. Note that R_3_ becomes clearly visible at *m/z* ≈ 5.15 when [P11] ≥ 1.7 µM.

**
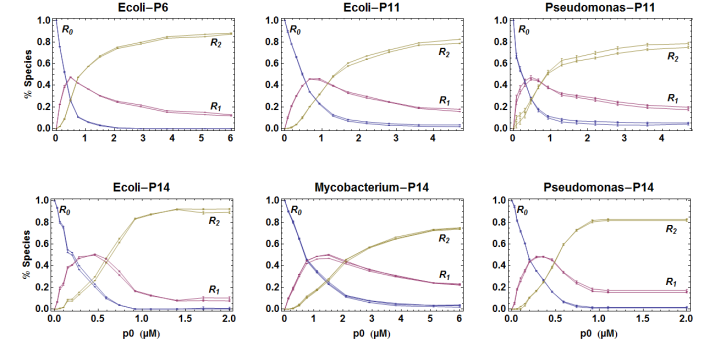
**

**Fig. S5A Variability of the fractions of species from different charge states**

This shows the variability of the fractions of species determined from the best spectra with different charge states. There were two such spectra in general, and three for Mycobacteium-P14 (which is not visible at the scale of the figure).

**
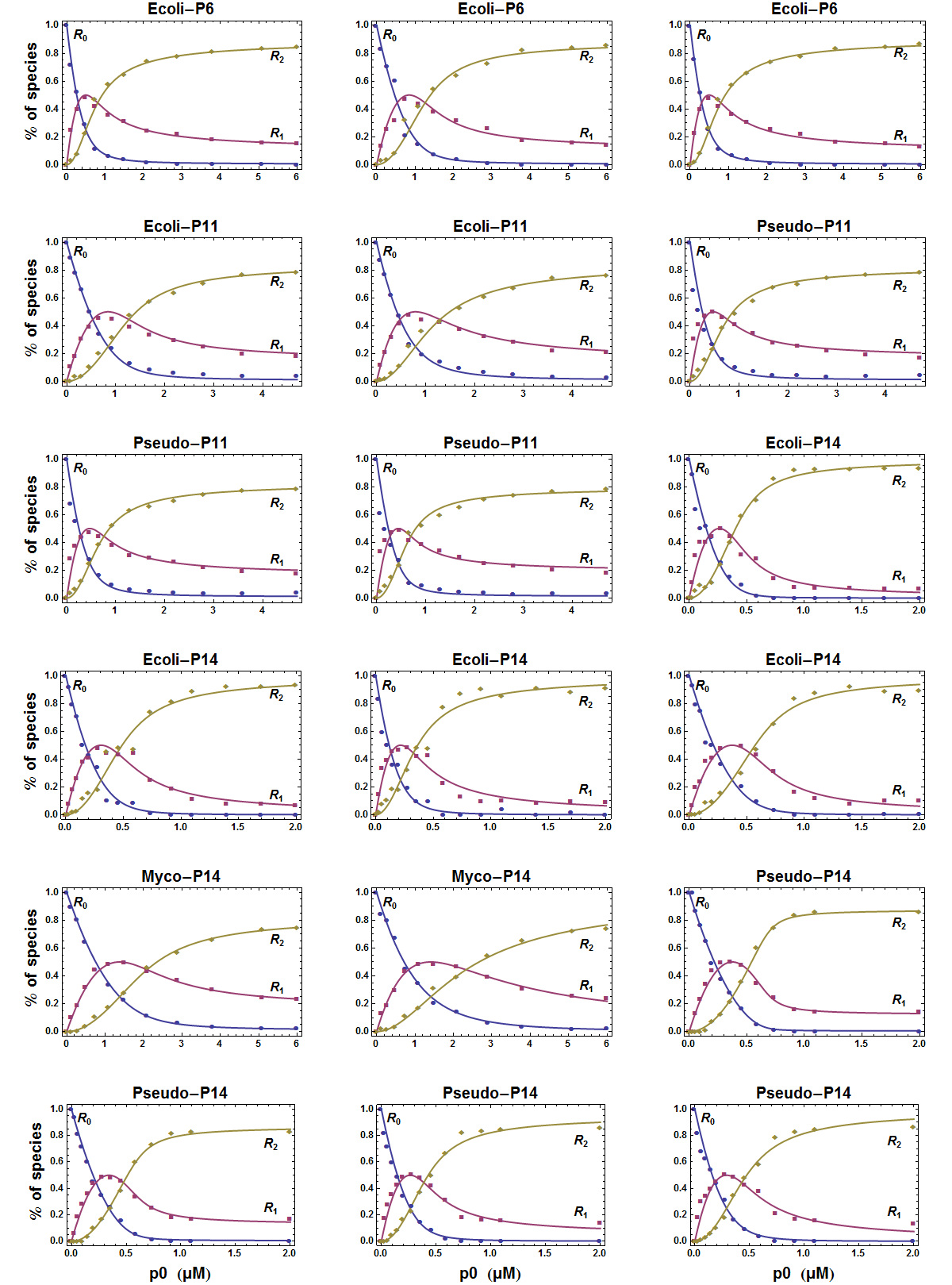
**

**Fig. S5B Complete panel of titration curves obtained by ESI-MS**


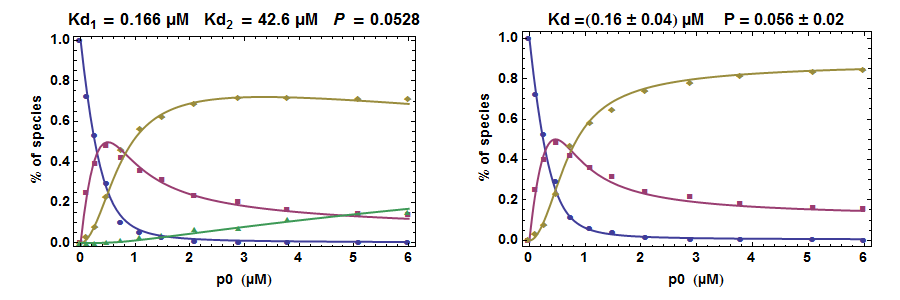


**Fig. S5C Characterization of the weak nonspecific interaction for β*_E. coli_*/P6**

The left/right panels show respectively the results from data processing taking/not taking into account separately the species(green ascending line + triangles in the left panel). It is seen that the values Kd_1_ = 0.166 µM and P = 0.0528 (left panel) are very close to$K_{d}$= 0.16 µM and P = 0.056 (right panel) due to the much lower affinity (Kd_2_ >> Kd_1_) of the nonspecific binding site(s).

**
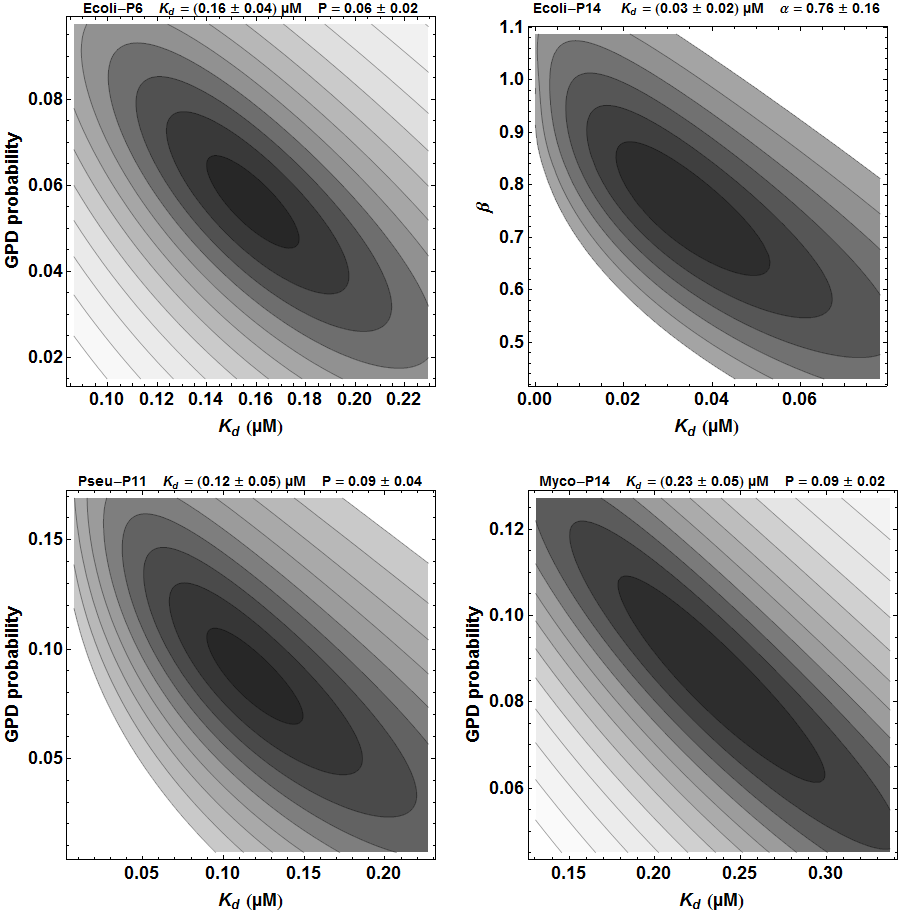
**

**Fig. S6 Analysis of parameter errors and correlations**

The logarithm of the residual sum of squares (SOS) minimized in the nonlinear regression procedure is represented as a 2D map with the two parameters used in the minimization being varied around their best values. These examples correspond to the subset of experiments shown in Fig. 5. The SOS increases from black to white. The two variable parameters are usually $K_{d}$and the probability$P$of GPD and, in some cases, when the best value for$P$was zero, the effective peptide fraction ***α*** was used as the second parameter. The latter parameter can be seen as a measure of the errors on the peptide and/or protein concentrations. In all situations, the iso-SOS ellipses are elongated along the descending diagonal. The different contour lines are separated by 0.25, which represents an increase of the SOS by a multiplicative factor of 1.284 between two successive contour lines.


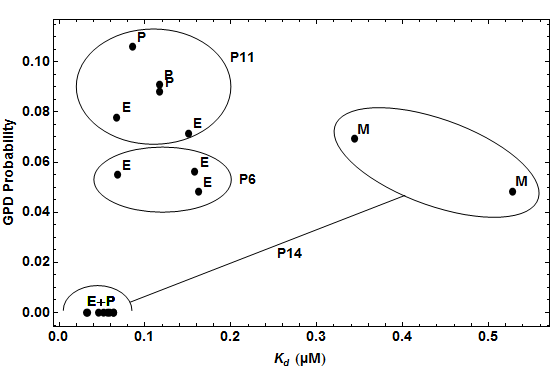


**Fig. S7 Lack of correlation between GPD and peptide affinity**

The graph shows the distribution of points with coordinates (K_d_ , GPD probability). Each point corresponds to a separate experiment for a given protein-peptide pair (contrary to Fig. 6 where the results for all separate experiments for a given protein-peptide pair were averaged). The ellipses group the results for a given peptide (**P6**, **P1**1 or **P14**) and each point is marked with E, M or P for, respectively, *E. coli*, *M. tuberculosis*, *P. aeruginosa*. The points for **P14** in the lower left part are too close from one another to be separated individually. Overall, the results highlight a clear segregation according to the nature of the peptide (apart for **P14**) and a total lack of correlation between K_d_ and GPD probability.


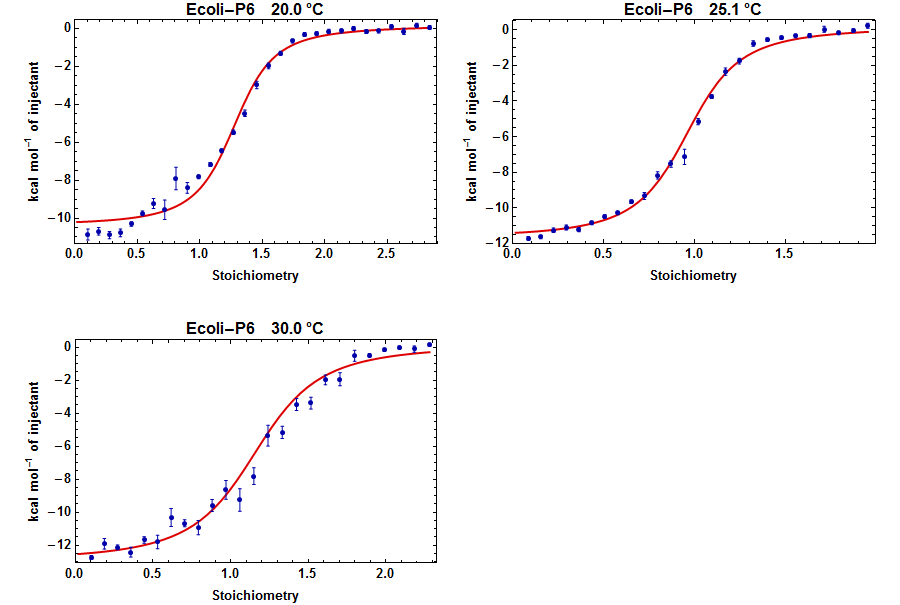


**
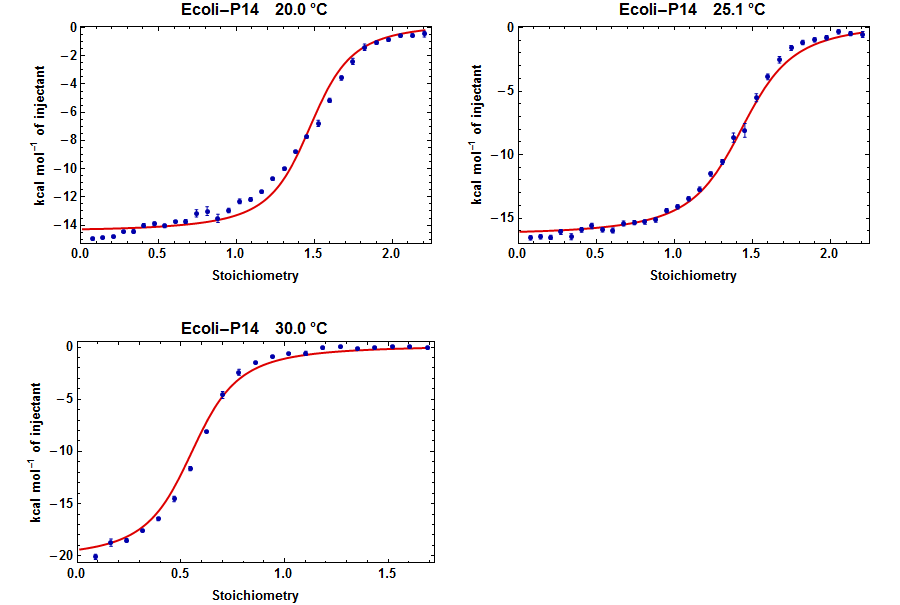
**

**
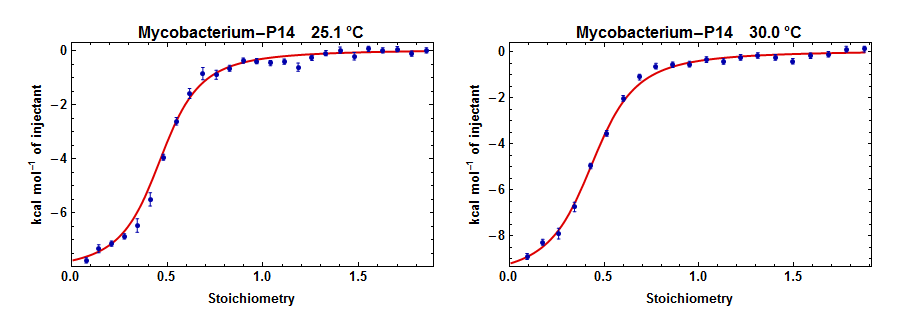
**

**
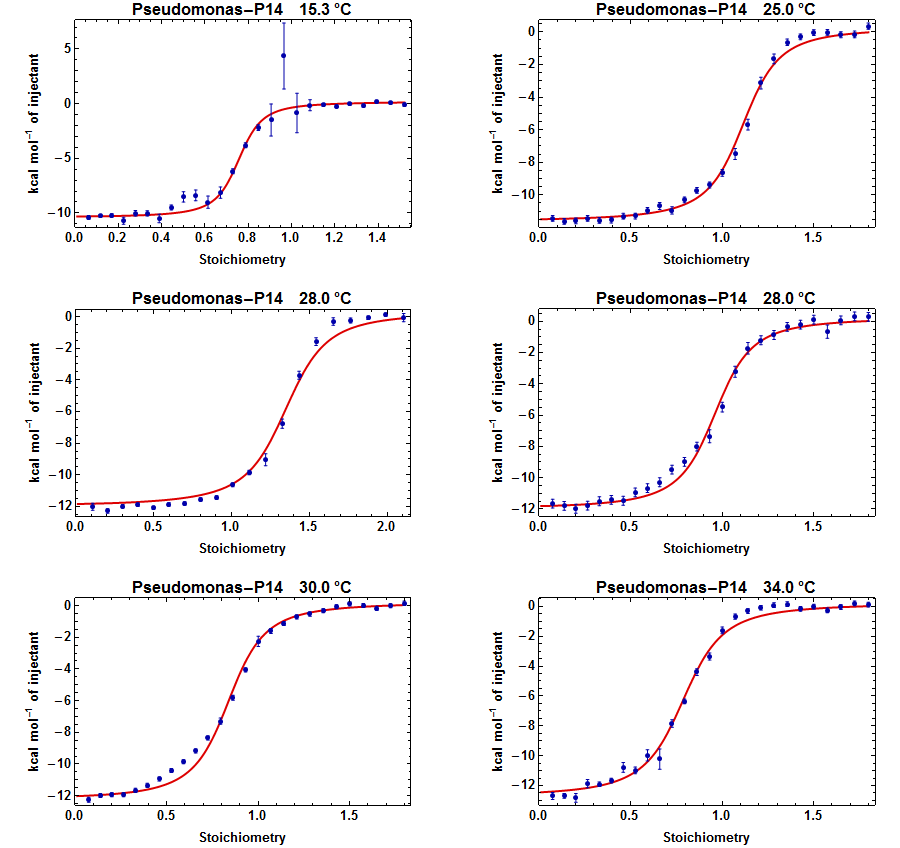
**

**Fig. S8 Global Thermodynamic Treatment of ITC titration curves**

All titration curves obtained at different temperatures for a protein-peptide pair were processed at once as described in [2]. Note that this ‘Global Thermodynamic Treatment’ seeks to explain several experimental curves with a minimum set of free parameters, which explains certainly the often not-so-good fit, **but only in part**. Indeed, the often visible lack of fit (*e.g.* for β*_E.coli_*/**P14** at 20 °C, of for β*_Pseudomonas_*/**P14** at 28 and 30 °C around a stoichiometric ratio of 0.6-0.7) is a mark of the simple association model being used accounting incompletely for the data. See Fig. S9 for results obtained after reprocessing of these data with two modes of binding.


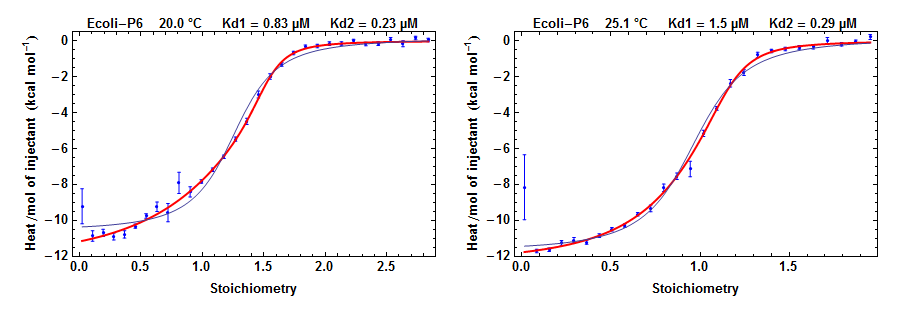


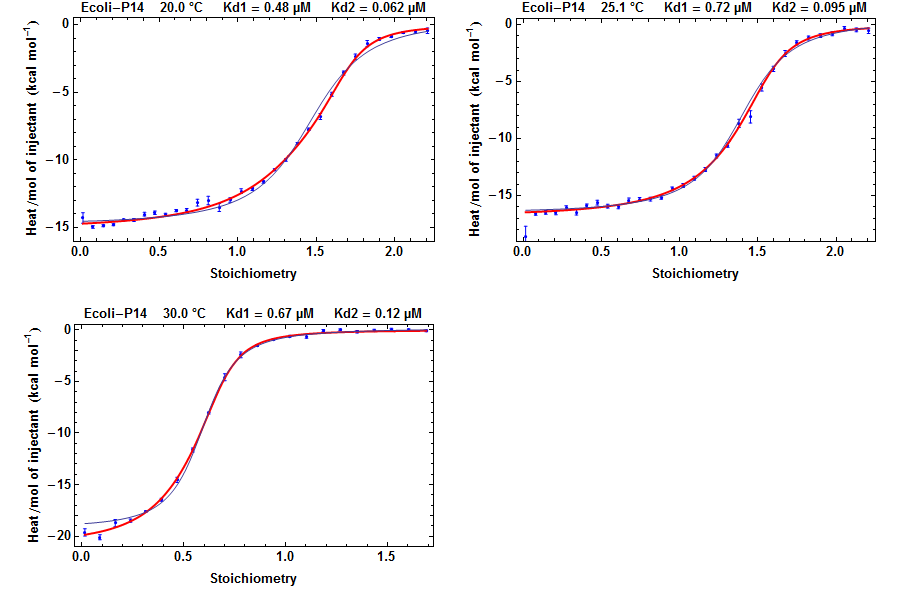


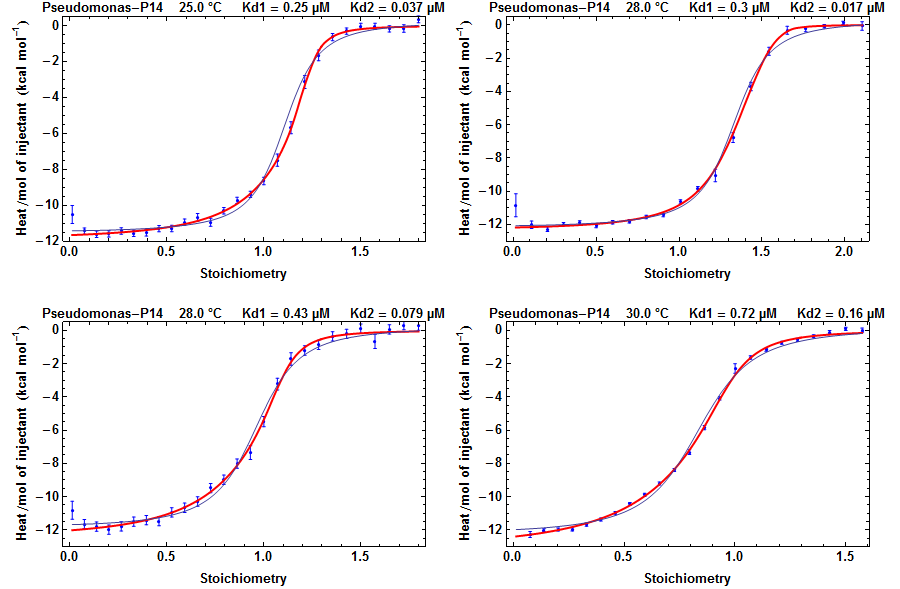


**Fig. S9 Individual treatment of ITC curves with two competing modes of binding**

Reprocessing of each individual titration curve with two modes of binding to the same site (red curves). The thin blue curves correspond to the best fit obtained with the single binding-mode (as in Fig. S8). Here, each titration curve was processed individually, whereas they were processed at once with the ‘Global Thermodynamic Treatment’ in Fig. S8. Only the titration curves leading to significant improvement of the fit are shown. All results are in Fig. S10.


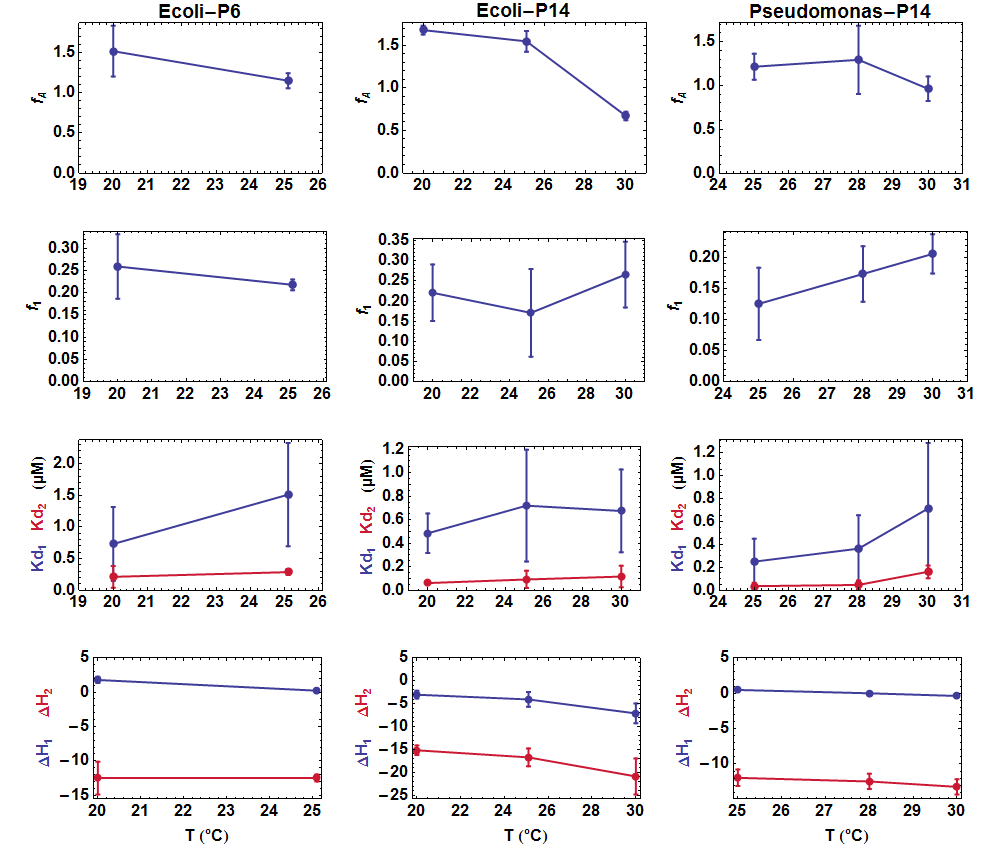


**Fig. S10 Parameters from ITC data processing with two modes of binding**

Evolution with the temperature of all parameters: f_A_ = ‘effective’ fraction of beta ring (which may account for concentration errors and/or incomplete dilution of the peptides), f_1_ = fraction of the first mode of binding (with parameters close to those after GTT in Fig. S8), f_2_ = 1 - f_1_ = fraction of the second mode of binding (not shown), and (Kd_1_ , ΔH_1_), (Kd_2_ , ΔH_2_) the dissociation constants and enthalpy variations for the first and second mode of binding, respectively (ΔH_1_ and ΔH_2_ are in kcal mol^-1^). The values at 28 °C for β*_Peudomonas_*-**P14** are the average of two experiments.


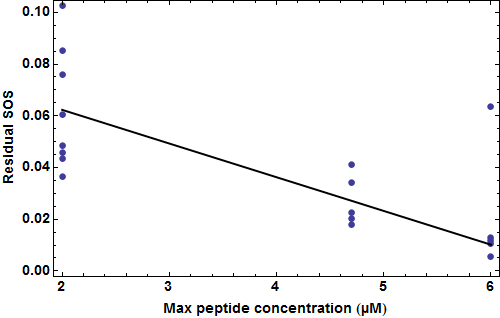


**Fig. S11 Experimental parameters and accuracy of the results**

The residual sum of squares (SOS) of the fit of all titration curves obtained by ESI-MS is shown as a function of the maximum concentration of the peptide used in the titration experiment. Apart for a very clear outlier at 6 µM (corresponding to an experiment with β*_E.coli_*/**P6**), there is a marked decrease of the SOS upon an increase of the maximum peptide concentration used in the titration. This stems from the fact that higher peptide concentration required to dispense larger volume of the stock peptide solution, and that the larger the dispensed volume, the higher the relative accuracy on the final peptide concentration (see text).

**References**

1. Sannes-Lowery, K.A., Griffey, R.H., Hofstadler, S.A.: Measuring dissociation constants of RNA and aminoglycoside antibiotics by electrospray ionization mass spectrometry. Anal Biochem. **280**, 264-271 (2000)

2. Burnouf, D., Ennifar, E., Guedich, S., Puffer, B., Hoffmann, G., Bec, G., et al.: kinITC: a new method for obtaining joint thermodynamic and kinetic data by isothermal titration calorimetry. J Am Chem Soc. **134**, 559-565 (2012)
